# PDGFD switches on stem cell endothelial commitment

**DOI:** 10.1007/s10456-022-09847-4

**Published:** 2022-07-20

**Authors:** Weisi Lu, Peipei Xu, Boxiong Deng, Jianing Zhang, Ying Zhan, Xianchai Lin, Xiangzhong Xu, Zhaoxia Xia, Xiaoxi Yang, Xiaoling Zeng, Lijuan Huang, Bingbing Xie, Chenghu Wang, Shasha Wang, Haiqing Kuang, Xianjing Han, Antonio Mora, Yihai Cao, Qin Jiang, Xuri Li

**Affiliations:** 1 State Key Laboratory of Ophthalmology, Zhongshan Ophthalmic Center, Sun Yat-sen University, Guangdong Provincial Key Laboratory of Ophthalmology and Visual Science, Guangzhou, 510060 China; 2 Affiliated Eye Hospital of Nanjing Medical University, Nanjing, 210000 China; 3 Department of Ophthalmology, The Sixth Affiliated Hospital of Sun Yat-sen University, Guangzhou, China; 4 Joint School of Life Sciences, Guangzhou Medical University and Guangzhou Institutes of Biomedicine and Health (Chinese Academy of Sciences), Guangzhou, China; 5 Department of Microbiology, Tumor and Cell Biology, Karolinska Institute, 17177 Stockholm, Sweden

**Keywords:** PDGFD, Stem cell, Endothelial commitment, Vascular differentiation

## Abstract

The critical factors regulating stem cell endothelial commitment and renewal remain not well understood. Here, using loss- and gain-of-function assays together with bioinformatic analysis and multiple model systems, we show that PDGFD is an essential factor that switches on endothelial commitment of embryonic stem cells (ESCs). PDGFD genetic deletion or knockdown inhibits ESC differentiation into EC lineage and increases ESC self-renewal, and PDGFD overexpression activates ESC differentiation towards ECs. RNA sequencing reveals a critical requirement of PDGFD for the expression of vascular-differentiation related genes in ESCs. Importantly, PDGFD genetic deletion or knockdown increases ESC self-renewal and decreases blood vessel densities in both embryonic and neonatal mice and in teratomas. Mechanistically, we reveal that PDGFD fulfills this function via the MAPK/ERK pathway. Our findings provide new insight of PDGFD as a novel regulator of ESC fate determination, and suggest therapeutic implications of modulating PDGFD activity in stem cell therapy.

**Supplementary Information:**

The online version contains supplementary material available at 10.1007/s10456-022-09847-4.

## Introduction

The platelet-derived growth factor (PDGF) family are known for their effects on mesenchyme and mural cells. Currently, there are four PDGFs (PDGFA, PDGFB, PDGFC, and PDGFD) and two receptors (PDGFR-α and PDGFR-β) [1]. PDGFD is a relatively new member of the family discovered many years after the finding of PDGFA and PDGFB, and binds to and activates PDGFR-β [1]. PDGFD has been shown to regulate fibrosis, inflammation, and tumorigenesis [2–4]. PDGFD has been reported to promote angiogenesis [5]. PDGFD is highly expressed in vascular cells in both mouse embryos and adults [6, 7], suggesting important roles of PDGFD in the vascular system. *Pdgfd* deficient mice are viable but display high blood pressure and disorganized pericytes in the cardiac vasculature [6], further suggesting a role of PDGFD in the regulation of the vascular system.

Embryonic stem cells (ESCs) are derived from the inner cell mass (ICM) of blastocyst-stage embryos and have the capacity for self-renewal and pluripotency. Therefore, ESCs may have great therapeutic potential in regenerative medicine. Cardiovascular diseases, such as cardiac infarction, heart failure, and artery diseases, are the leading cause of mortality and morbidity worldwide [8]. Such diseases are often related to impaired vascular endothelial cell (EC) function. As such, derivation of functional and healthy ECs from ESCs has been a focus of interest in the field [9]. Recent advances in single cell RNA sequencing have provided new insight into stem cell-derived vascular cells [10]. Yet, critical molecules and mechanisms regulating ESC differentiation towards vascular cells remain poorly understood. Also, although PDGFD is expressed during embryonic development [7], it remains thus far unknown whether PDGFD plays a role in ESC regulation.

Here, we show that PDGFD is a critical factor that turns on ESC endothelial commitment. *Pdgfd* knockdown in ESCs increased ESC self-renewal and inhibited ESC differentiation into endothelial lineage in multiple assays. RNA sequencing analysis revealed that *Pdgfd* depletion caused transcriptome-wide downregulation of differentiation-related genes, particularly, vascular EC-related genes. Importantly, genetic deletion of *Pdgfd* in mice decreased cardiac blood vessel density in both embryonic and neonatal mice, and PDGFD knockdown in ESCs decreased blood vessel density during teratoma formation. Mechanistically, PDGFD fulfilled its function in ESCs via the mitogen-activated protein kinase/extracellular signal-regulated kinase (MAPK/ERK) pathway, and blocking MAPK/ERK signaling diminished PDGFD’s effect on ESCs. Collectively, our findings provide new insights into the mechanisms regulating ESC differentiation and vascular EC commitment, and suggest that modulating PDGFD activity may have therapeutic value in stem cell therapy.

## Results

### PDGFD is upregulated upon ESC differentiation

**Fig. 1:**
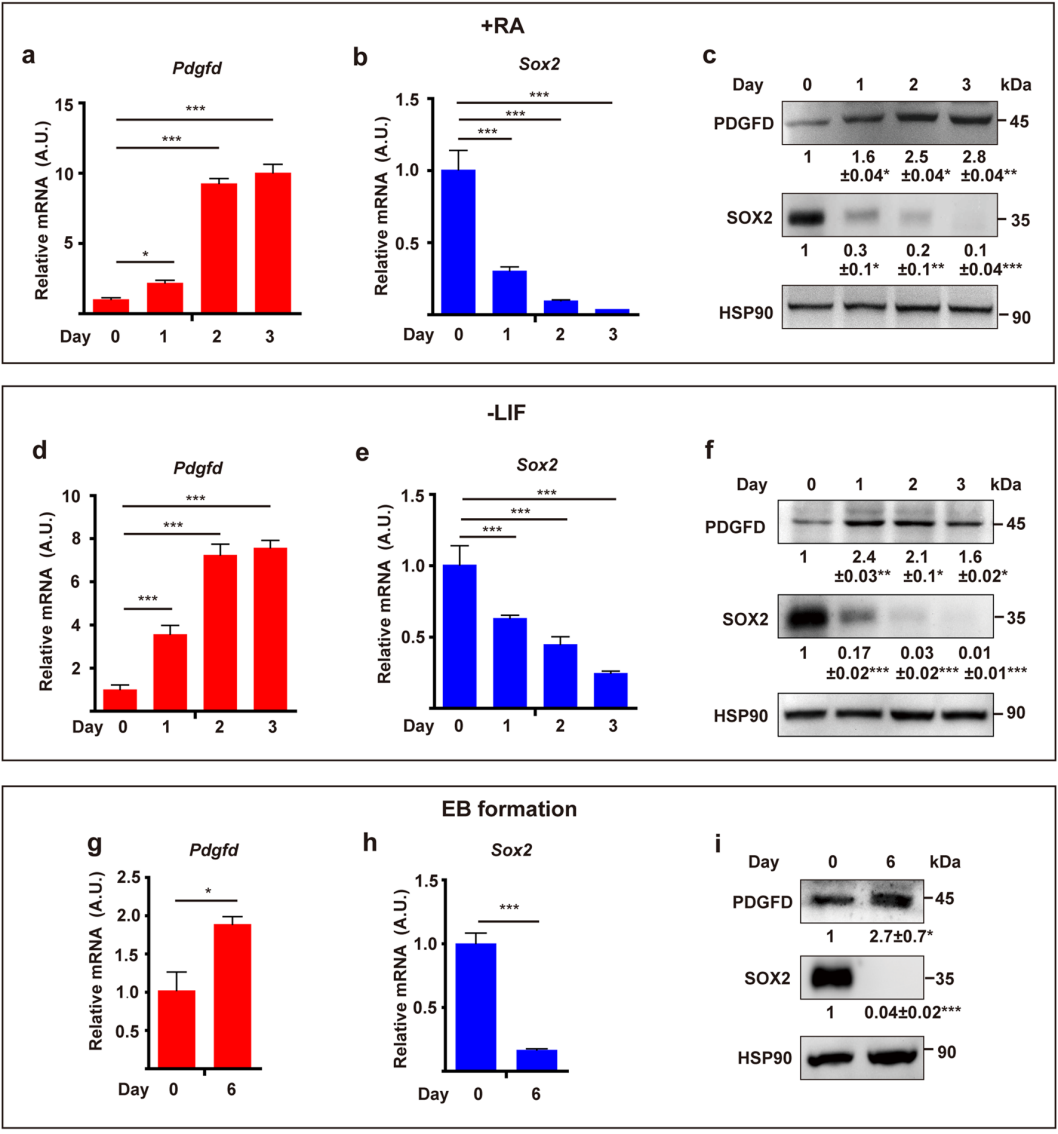
PDGFD is expressed in ESCs and upregulated upon ESC differentiation. **a**–**c** qRT-PCR (**a**, **b**) and Western blot (**c**) analyses showing PDGFD and SOX2 expression during ESC differentiation induced by retinoic acid treatment (+ RA). **d**–**f** qRT-PCR (**d**, **e**) and Western blot (**f**) analyses showing PDGFD and SOX2 expression during ESC differentiation induced by leukemia inhibitory factor withdrawal (−LIF). **g**–**i** qRT-PCR (**g**, **h**) and Western blot (**i**) analyses of PDGFD and SOX2 expression during ESC differentiation induced by embryoid body (EB) formation. In the experiments depicted in **c**, **f**, and **i**, densitometric quantification normalized to HSP90 and expressed relative to day 0 is shown beneath the blots. All data are presented as mean ± SD, *n* = 3 each group. Statistical significance was determined using one-sample *t*-test in **c**, **f**, **i** and one-way ANOVA in **a**, **b**, **d**, **e**, **g**, **h**. *p < 0.05, **p < 0.01, ***p < 0.001

PDGFD is expressed during embryonic development [7]. Yet, it remains thus far unknown whether PDGFD plays a role in ESC regulation. We found that PDGFD expression in ESCs increased markedly upon ESC differentiation in multiple ESC differentiation assays, such as ESC differentiation induced by retinoic acid treatment (+RA), withdrawal of leukemia inhibitory factor (−LIF) and embryonic body (EB) formation. Both qRT-PCR (Fig. 1a, d, g) and Western blots (Fig. 1c, f, i) showed gradual increases of PDGFD transcript and protein levels during ESC differentiation with concomitant loss of SOX2, a key pluripotency factor (Fig. 1b, c, e, f, h, i), suggesting a potential effect of PDGFD on ESC differentiation. PDGFD is proteolytically cleaved by plasmin or uPA [11, 12], and cleaved forms of PDGFD were detected in ESC-conditioned serum-free medium with or without LIF by Western blot (Fig. S1a). Consistently, both plasmin and uPA were found during ESC differentiation under various conditions (Fig. S1b–d).

### PDGFD promotes ESC differentiation and inhibits ESC self-renewal

**Fig. 2:**
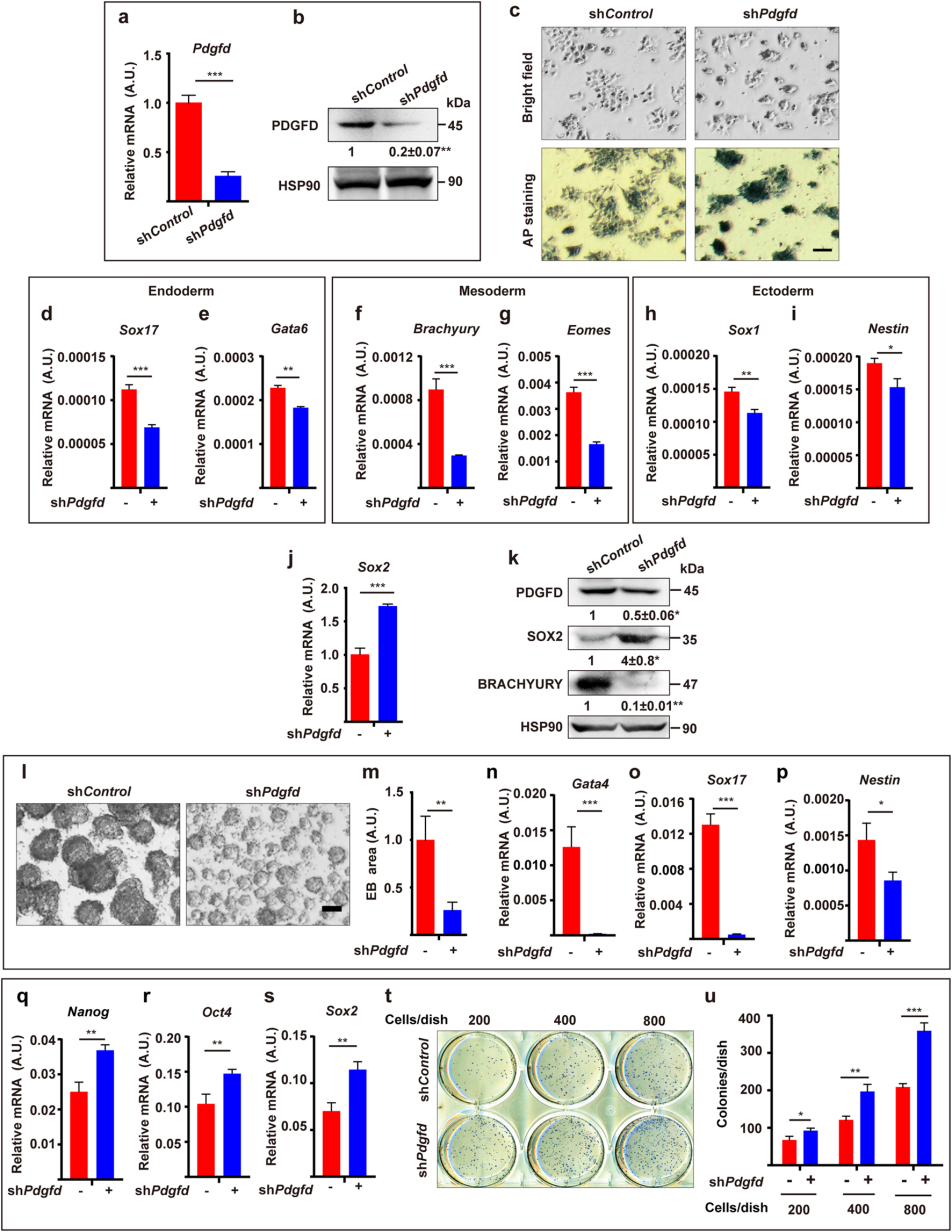
PDGFD promotes ESC differentiation and inhibits ESC self-renewal. **a**, **b** qRT-PCR (**a**) and Western blot (**b**) showing PDGFD expression in ESCs with *Pdgfd* knockdown (sh*Pdgfd*). **c** Images showing sh*Pdgfd* ESCs cultured in the absence of LIF and after AP staining (lower panel). *Scale bar* 500 μm. **d**–**k** qRT-PCR (**d**–**j**) and Western blot (**k**) showing the expression of differentiation markers (**d**–**i**) and *Sox2* (**j**, **k**) in sh*Pdgfd* ESCs. **l**, **m** Shown are EB formation of sh*Pdgfd* ESCs. EBs were imaged (**l**) and EB areas quantified (**m**). *Scale bar* 100 μm. *AU* arbitrary unit. **n**–**p** qRT-PCR analysis of the expression of differentiation markers in EBs derived from sh*Pdgfd* ESCs. **q**–**s** PDGFD knockdown upregulated the key ESC pluripotent markers Nanog, Oct4 and Sox2 in an EB formation assay as shown by qRT-PCR. **t**, **u** Results showing AP^+^ colonies of sh*Control* or sh*Pdgfd* ESCs seven days after plating at different densities. In the experiments depicted in b and k, densitometric quantification normalized to HSP90 and expressed relative to sh*Control* is shown beneath the blots. Data are presented as mean ± SD, *n* = 3 each group. Statistical significance was determined using one-sample *t*-test in **b**, **k** and Student’s *t*-test in **a**, **d**–**j**, **m**–**s**, **u**. *p < 0.05, **p < 0.01, ***p < 0.001

To investigate the potential effect of PDGFD on ESCs, we knocked down *Pdgfd* (sh*Pdgfd*) in mouse ESCs, which was confirmed by qRT-PCR and Western blot (Fig. 2a, b). We found that PDGFD knockdown inhibited LIF withdrawal-induced ESC differentiation as shown by stronger AP staining, more compact colonies compared with control ESCs (sh*Control*, Fig. 2c), decreased expression of many differentiation markers of the three germ layers and increased expression of *Sox2*, a key pluripotency marker (Fig. 2d–k). In addition, sh*Pdgfd* ESCs formed significantly smaller EBs (Fig. 2l, m) with downregulation of many differentiation markers, such as *Gata4*, *Sox17*, and *Nestin* (Fig. 2n–p) in an EB formation-induced ESC differentiation assay, demonstrating that PDGFD is critically required for ESC differentiation. In addition, in an EB formation assay, PDGFD knockdown upregulated the key ESC pluripotent markers *Nanog*, *Oct4* and *Sox2* as shown by qRT-PCR (Fig. 2q–s), suggesting increased ESC self-renewal. Indeed, ESCs with PDGFD knockdown formed more colonies in a colony formation assay (Fig. 2t, u). Moreover, the enhanced ESC self-renewal was further supported by the increased ESC proliferation as shown by the greater number of ESCs at different time points after plating (Fig. S2a). Together, these data demonstrate PDGFD induces ESC differentiation and inhibits ESC self-renewal.

To verify the specificity of the effect of PDGFD, we also investigated whether PDGFB had the same effect compared with PDGFD, since like PDGFD, PDGFB also binds to PDGFR-β [1]. By contrast to PDGFD, the knockdown of which increased ESC proliferation (Fig. S2a), *Pdgfb* knockdown slightly decreased ESC proliferation instead (Fig. S2b). Moreover, opposite to PDGFD, who’s knockdown up-regulated the pluripotency markers Nanog, Oct4 and Sox2 (Fig. 2q–s), *Pdgfb* knockdown decreased their expression instead (Fig. S2c–f), demonstrating that the effect of PDGFD was specific.

### PDGFD is required for the expression of vascular-differentiation-related genes in ESCs

**Fig. 3:**
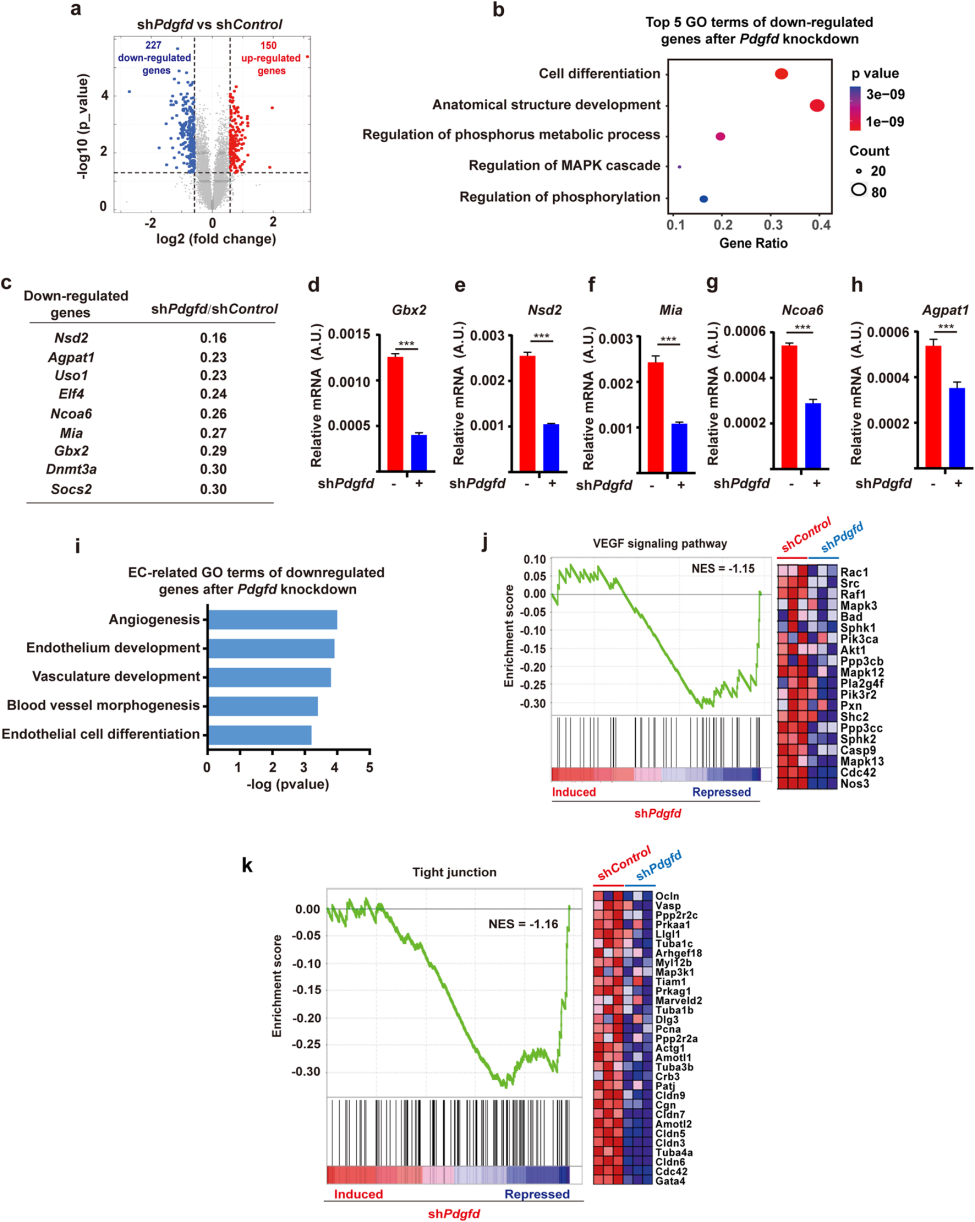
PDGFD is essential for the expression of vascular-differentiation-related genes in ESCs. **a** A volcano plot of RNA sequencing of sh*Pdgfd* ESCs showing 150 up- and 227 down-regulated genes (*p* < 0.05). **b** Gene Ontology (GO) analysis showing the top five GO terms of genes downregulated in sh*Pdgfd* ESCs. The dot size represents the number of differentially expressed genes of the GO term. **c** Top ten differentiation-related genes downregulated in sh*Pdgfd* ESCs. **d**–**h** qRT-PCR results showing the downregulation of the differentiation-related genes in sh*Pdgfd* ESCs. Data are presented as mean ± SD, *n* = 3 each group. Statistical significance was determined using Student’s *t*-test. ***p < 0.001. **i** GO analysis showing the differentially expressed genes related to the vascular system in sh*Pdgfd* ESCs. **j**, **k** Gene set enrichment analysis (GSEA) depicting enrichment of VEGF signaling pathway (**j**) and tight junction (**k**)-related gene sets in sh*Pdgfd* ESCs. *NES* normalized enrichment score

To investigate the genes regulated by PDGFD, we performed RNA sequencing using sh*Pdgfd* ESCs, and found 150 up- and 227 down-regulated genes in sh*Pdgfd* ESCs (> 1.5-fold change, *p*-value < 0.05, Fig. 3a). Gene Ontology (GO) analysis showed that the down-regulated genes in sh*Pdgfd* ESCs were mostly related to cell differentiation (Fig. 3b), such as *Nsd2*, *Agpat1*, *Uso1*, *Elf4*, *Ncoa6*, *Mia*, *Gbx2*, *Dnmt3a*, and *Socs2* (Fig. 3c), and many of the up-regulated genes were linked to ESC pluripotency (Fig. S3a). The RNA sequencing results were confirmed by qRT-PCR (Fig. 3d–h). Importantly, GO term analysis revealed that many of the genes downregulated in sh*Pdgfd* ESCs were vascular-related, such as genes related to “angiogenesis”, “endothelium development’, “vasculature development”, “blood vessel morphogenesis” and “endothelial cell differentiation” (Fig. 3i), suggesting a role of PDGFD in these processes. In line with this, gene set enrichment analysis (GSEA) also unveiled that *Pdgfd* knockdown suppressed genes related to vascular endothelial-derived growth factor signaling pathways or tight junctions (Fig. 3j, k), both of which are essential for vascular development and function. These data thus demonstrate that PDGFD is critically required for the expression of vascular-differentiation-related genes in ESCs.

### PDGFD promotes endothelial cell (EC) commitment of ESCs

**Fig. 4:**
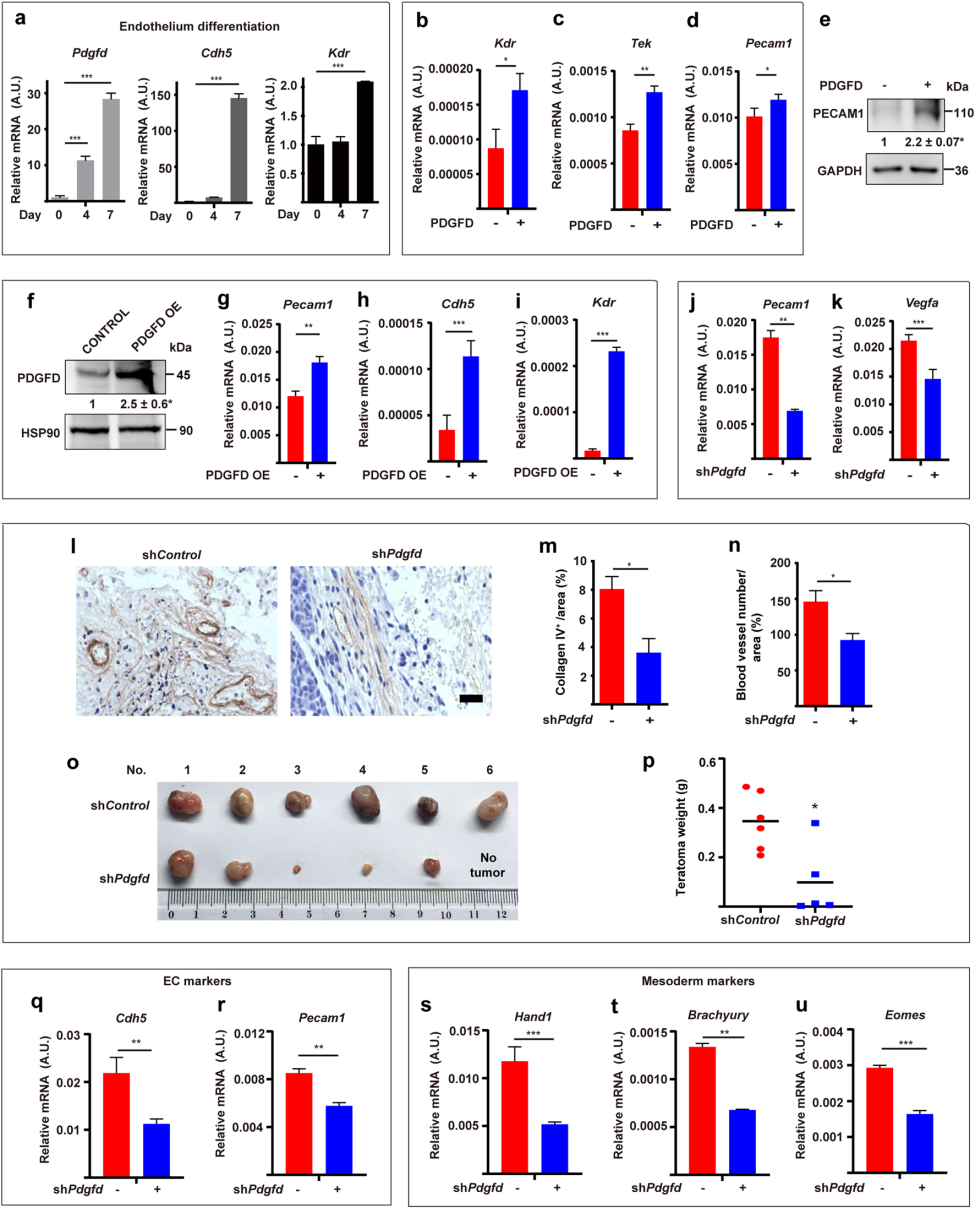
PDGFD promotes ESC endothelial commitment. qRT-PCR results showing the expression of *Pdgfd* and endothelial markers (*Cdh5* and *Kdr*) during endothelium differentiation of ESCs. **b**–**e** qRT-PCR (**b**–**d**) and Western blot (**e**) results showing that PDGFD protein treatment increased the expression of endothelial markers in ESCs (no VEGFA treatment). **f**–**i** Western blot (**f**) and qRT-PCR (**g**–**i**) results showing that overexpression of PDGFD (PDGFD OE) increased the expression of endothelial marker genes in ESCs (no VEGFA treatment). **j**, **k** qRT-PCR results showing decreased expression of endothelial marker genes in sh*Pdgfd* ESCs (no VEGFA treatment). **l**–**n** Immunohistochemistry staining for collagen IV was performed to identify blood vessels in the teratomas derived from sh*Control* or sh*Pdgfd* ESCs (**l**). Quantification of collagen IV density and vessel numbers per field are shown in m and n. *Scale bar* 50 μm. **o**, **p** Shown are teratomas (**o**) and their weights (**p**) derived from sh*Control* and sh*Pdgfd* ESCs. **q**–**u** qRT-PCR results showing the expression of endothelial cell (EC) markers (**q**, **r**) and mesoderm markers (**s**–**u**) of sh*Control* and sh*Pdgfd* teratomas. Densitometric quantification normalized to GAPDH (**e**), HSP90 (**f**) and expressed relative to Control group is shown beneath the blots. Data are presented as mean ± SD, *n = *3 each group. Statistical significance was determined using one-sample *t*-test in **e**, **f** and one-way ANOVA in **a** and Student’s *t*-test in **b**–**d**, **g**–**k**, **m**, **n**, **p**–**u**. *p < 0.05, **p < 0.01, ***p < 0.001

Led by our findings of the effect of PDGFD on the expression of vascular-related genes in ESCs, we subsequently investigated whether PDGFD plays a role in ESC differentiation into vascular ECs. In a VEGFA-induced EC differentiation assay, we found a marked upregulation of *Pdgfd* together with the EC markers *Cdh5* and *Kdr*, with *Pdgfd* upregulation being earlier (Fig. 4a), suggesting a causative role of PDGFD in EC differentiation of ESCs. Consistently, in a VEGFA-induced EC differentiation assay, *Pdgfd* knockdown dampened the expression of the EC markers *Cdh5* and *Kdr* (Fig. S3b, c). Moreover, PDGFD protein treatment of ESCs (Fig. 4b–e) or PDGFD overexpression (PDGFD OE, Fig. 4f–i) markedly increased the expression of EC markers, while *Pdgfd* knockdown decreased their expression (Fig. 4j, k). Different from PDGFD, who’s treatment up-regulated EC markers in ESCs, PDGFB treatment did not display such an effect (Fig. S2g, h). Noteworthy, these findings are consistent with previous reports showing different effects of PDGFD and PDGFB on adipose-derived stem cells [13] and support that the effects of PDGFD on ESCs were specific. Importantly, in a teratoma formation assay in vivo, *Pdgfd* knockdown decreased tumor blood vessel density and collagen IV expression (a marker for blood vessels) (Fig. 4l–n), with smaller tumor size and weight (Fig. 4o, p). Consistently, qRT-PCR showed decreased expression of EC markers (*Cdh5* and *Pecam1*, Fig. 4q, r) and mesoderm markers (*Hand*1, *Brachyury* and *Eomes*) in sh*Pdgfd-*depleted teratomas (Fig. 4s–u). Collectively, these data demonstrate an important role of PDGFD in promoting EC commitment of ESCs and blood vessel formation.

### MAPK/ERK signaling mediates the effects of PDGFD on ESCs

**Fig. 5:**
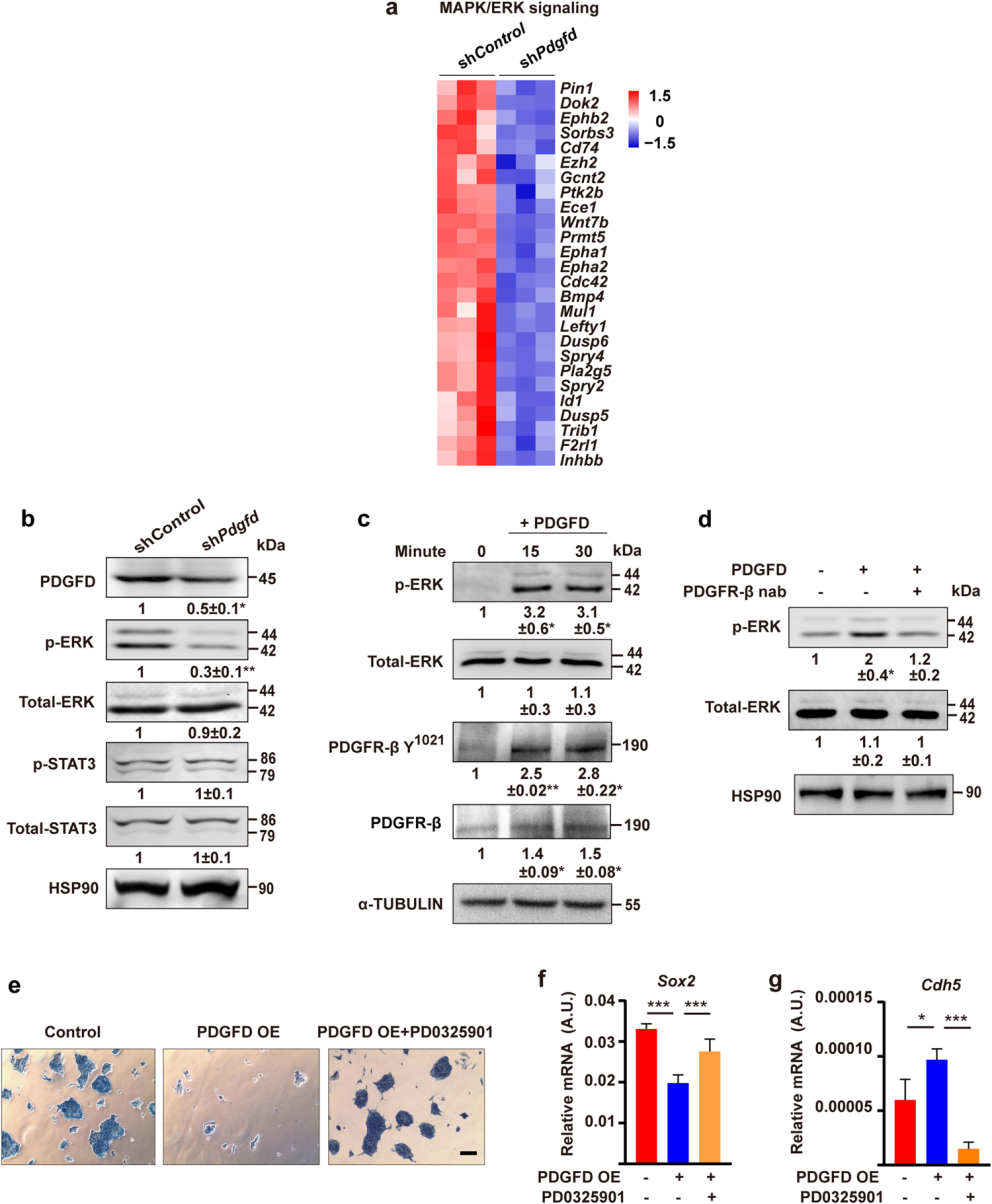
MAPK/ERK signaling and PDGFR-β mediate effects of PDGFD on ESCs. **a** Heatmap showing the differentially expressed genes in sh*Pdgfd* ESCs related to MAPK/ERK signaling. **b** Western blot result showing reduced ERK phosphorylation in sh*Pdgfd* ESCs while STAT3 phosphorylation was not changed. **c** Western blot result showing PDGFD-induced ERK and PDGFR-β phosphorylation as well as PDGFR-β upregulation in ESCs. **d** Western blot result showing that administration of PDGFR-β neutralizing antibody (nab) abolished PDGFD-induced ERK phosphorylation in ESCs. **e** AP staining showing that administration of an ERK pathway inhibitor PD0325901 abolished PDGFD overexpression (PDGFD OE)-induced inhibition of colony formation in ESCs. *Scale bar* 300 μm. **f**, **g** qRT-PCR results showing that administration of an ERK pathway inhibitor PD0325901 abolished PDGFD overexpression (PDGFD OE)-induced down-regulation of *Sox2* and up-regulation of *Cdh5* in ESCs. Densitometric quantification normalized to HSP90 (**b**, **d**) and α-TUBULIN (**c**) and expressed relative to Control group is shown beneath the blots. Data are presented as mean ± SD, *n* = 3 each group. Statistical significance was determined using one-way ANOVA in **f**, **g** and one-sample *t*-test in **b**–**d**. *p < 0.05, **p < 0.01, ***p < 0.001

We next investigated the signaling pathway induced by PDGFD in ESCs. RNA-seq results showed that numerous genes of the MAPK/ERK pathways were downregulated by *Pdgfd* knockdown (Fig. 5a), indicating important functions of them in PDGFD-induced effects. Consistently, Western blot showed that *Pdgfd* knockdown reduced ERK phosphorylation (p-ERK) in ESCs, while STAT3, a known PDGFD downstream effector [5], was not affected (Fig. 5b). Moreover, PDGFD protein treatment activated ERK and PDGFR-β and up-regulated PDGFR-β at different time points in ESCs (Fig. 5c), consistent with previous reports that PDGFRs can be up-regulated rapidly within 10 min [14–17]. Furthermore, administration of PDGFR-β neutralizing antibody (nab) (Fig. 5d) or a PDGFR inhibitor Crenolanib (Fig. S4a) abolished PDGFD-induced ERK activation, indicating an important role of PDGFR-β in mediating PDGFD’s effects. Importantly, administration of an ERK inhibitor PD0325901 completely abolished PDGFD overexpression (PDGFD OE, Fig. S4b)-induced inhibition of ESC colony formation, AP staining (Fig. 5e), downregulation of *Sox2*, and upregulation of EC marker *Cdh5* in ESCs (Fig. 5f, g). In addition, inhibition of ERK signaling by an ERK inhibitor PD0325901 up-regulated SOX2 (Fig. S4c) and down-regulated the EC markers *Kdr* and *Pecam1* (Fig. S4d, e), thus phenocopying the effect of PDGFD knockdown. The ERK inhibitor PD0325901-induced SOX2 up-regulation was confirmed by Western blot (Fig. S4f).

It is known that SOX2 translocation from nucleus to cytoplasm promotes ESC differentiation [18]. We then tested whether ERK activation regulated this process by treating the ESCs with an ERK activator 12-*O*-tetradecanoylphorbol-13-acetate (TPA). We found rapid ERK phosphorylation (Fig. S4g) with concomitant translocation of SOX2 from nucleus to cytoplasm as shown by the reduced nucleus and increased cytoplasmic SOX2 levels (Fig. S4g). These data suggest that PDGFD-induced ESC differentiation may be fulfilled by ERK activation-induced SOX2 translocation from nucleus to cytoplasm.

### Genetic deletion of *Pdgfd* impaired vascular endothelial cell development in mice

**Fig. 6:**
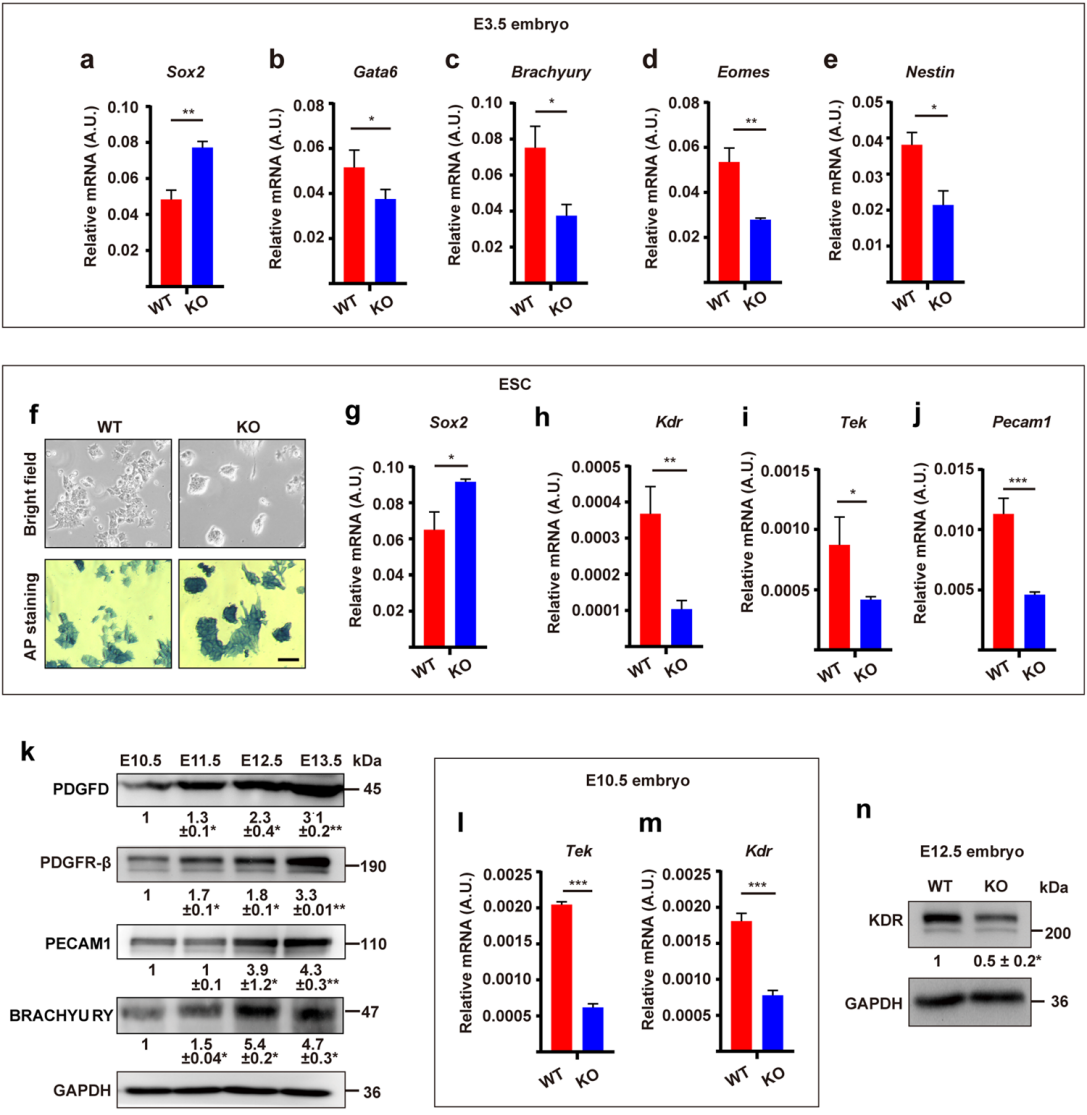
Genetic deletion of *Pdgfd* impairs ESC differentiation in vivo and in vitro. **a**–**e** qRT-PCR result showing the upregulation of *Sox2* and downregulation of differentiation-related genes in *Pdgfd* knockout (KO) E3.5 embryos. *WT* wild-type. **f**–**j** WT and *Pdgfd* KO ESCs were cultured without LIF and subjected to imaging (**f**, upper panel) and AP staining (**f**, lower panel). qRT-PCR results showing the upregulation of *Sox2* and downregulation of EC markers in **f** (**g**–**j**). *Scale bar* 500 μm. **k** Western blot results showing the expression levels of PDGFD, PDGFR-β, PECAM1, and BRACHYURY in mouse embryos from E10.5 to E13.5. **l**–**n** qRT-PCR (**l**, **m**) and Western blot (**n**) results showing the expression levels of EC markers in WT and *Pdgfd* KO embryos. Densitometric quantification normalized to GAPDH (**k**, **n**) and expressed relative to E10.5 and WT group, respectively, is shown beneath the blots. Data are presented as mean ± SD, *n* = 3 each group. Statistical significance was determined using one-sample *t-*test in **k**, **n** and Student’s *t*-test in **a**–**e**, **g**–**j**, **l**, **m**. *p < 0.05, **p < 0.01, ***p < 0.001

**Fig. 7:**
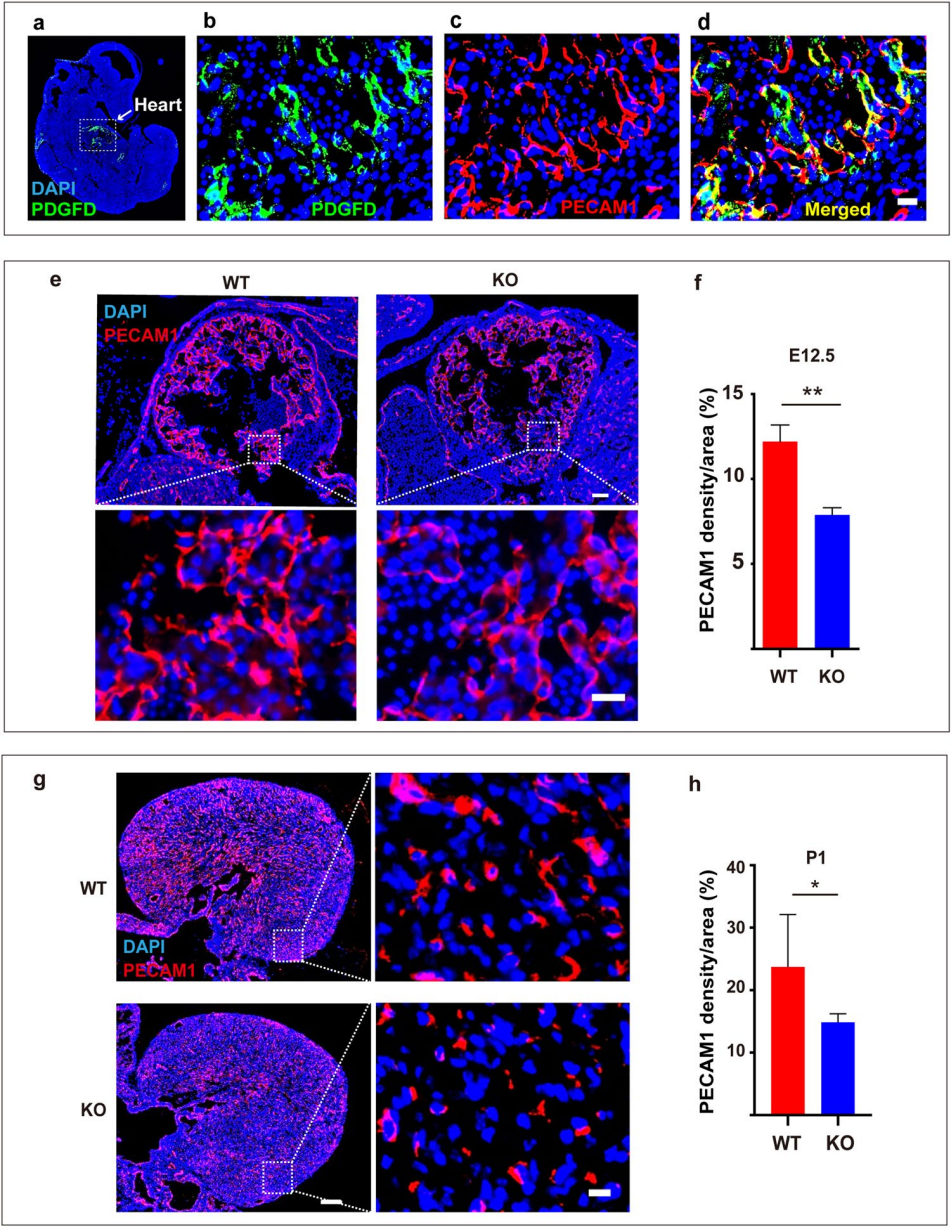
Loss of *Pdgfd* impairs vascular development in mice. **a**–**d** Immunofluorescence staining of PDGFD and PECAM1 in E12.5 mouse embryos. Shown in **a** is PDGFD staining on a section of a mouse embryo. Shown in **b**–**d** are high-magnification images of PDGFD and PECAM1 staining in the heart of an E12. 5 mouse embryo. *Scale bars* 20 μm. **e**, **f** Shown in e are PECAM1 immunofluorescence staining of hearts of E12.5 WT and *Pdgfd* KO embryos. Shown in **f** are the quantifications of **e**. *Scale bars* 100 μm on the top and 10 μm on the bottom. *n* = 4 for WT, and *n* = 5 for KO. **g**, **h** Shown in g are PECAM1 immunofluorescence staining of hearts of P1 WT and *Pdgfd* KO mice. Shown in **h** are the quantifications of **g**. *Scale bars* 200 μm on the left and 10 μm on the right. *n* = 5 for WT, and *n* = 6 for KO. Data are presented as mean ± SD, *n* = 3 each group. Statistical significance was determined using Student’s *t*-test in **f** and **h**. *p < 0.05, **p < 0.01

To verify the role of PDGFD in vivo, we generated *Pdgfd-*deficient mice (Fig. S5a–c). In embryonic day 3.5 (E3.5) embryos, from which ESCs are derived, *Pdgfd* deletion upregulated *Sox2* and downregulated many differentiation marker genes (Fig. 6a–e), suggesting increased ESC renewal and decreased ESC differentiation. Indeed, *Pdgfd* knockout (KO) ESCs derived from E3.5 embryos formed more compact colonies with stronger AP staining (Fig. 6f), higher *Sox2* expression levels and decreased expression of many EC markers, such as *Kdr*, *Tie2*, and *Pecam1* (Fig. 6g–j), further showing increased ESC renewal. The vascular system forms between E10.5 and E13.5 during embryonic development [19]. Noteworthy, the expression of PDGFD and PDGFR-β increased during this time course together with the EC marker PECAM1 (Fig. 6k), suggesting important roles of PDGFD in vascular development. By contrast to PDGFD, the protein levels of PDGFB decreased during E10.5–E13.5 instead (Fig. S6a), suggesting a different function of PDGFB. Indeed, scRNA-seq analysis of E9.5–10.5 mouse embryos [20] also revealed markedly higher expression levels of PDGFD than those of PDGFB in endothelium (Fig. S6b), further supporting an important and unique role of PDGFD in endothelium formation. Importantly, in both E10.5 and E12.5 *Pdgfd* KO embryos, the expression of many vascular markers decreased (Fig. 6l–n). Noteworthy, PDGFD was mainly detected in the heart of mouse embryos at E12.5 (Fig. 7a) and was co-localized with PECAM1^+^ cells (Fig. 7b–d), demonstrating that PDGFD is highly expressed in the developing cardiac vasculature. In line with the reduced EC markers in *Pdgfd*^−/−^ embryos, decreased blood vessel densities were found in the *Pdgfd*^−/−^ hearts at both E12.5 (Fig. 7e, f) and postnatal day 1 (P1) (Fig. 7g, h) as revealed by PECAM1 staining. Together, these data demonstrate that genetic deletion of *Pdgfd* impaired vascular EC development in vivo.

## Discussion

**Fig. 8:**
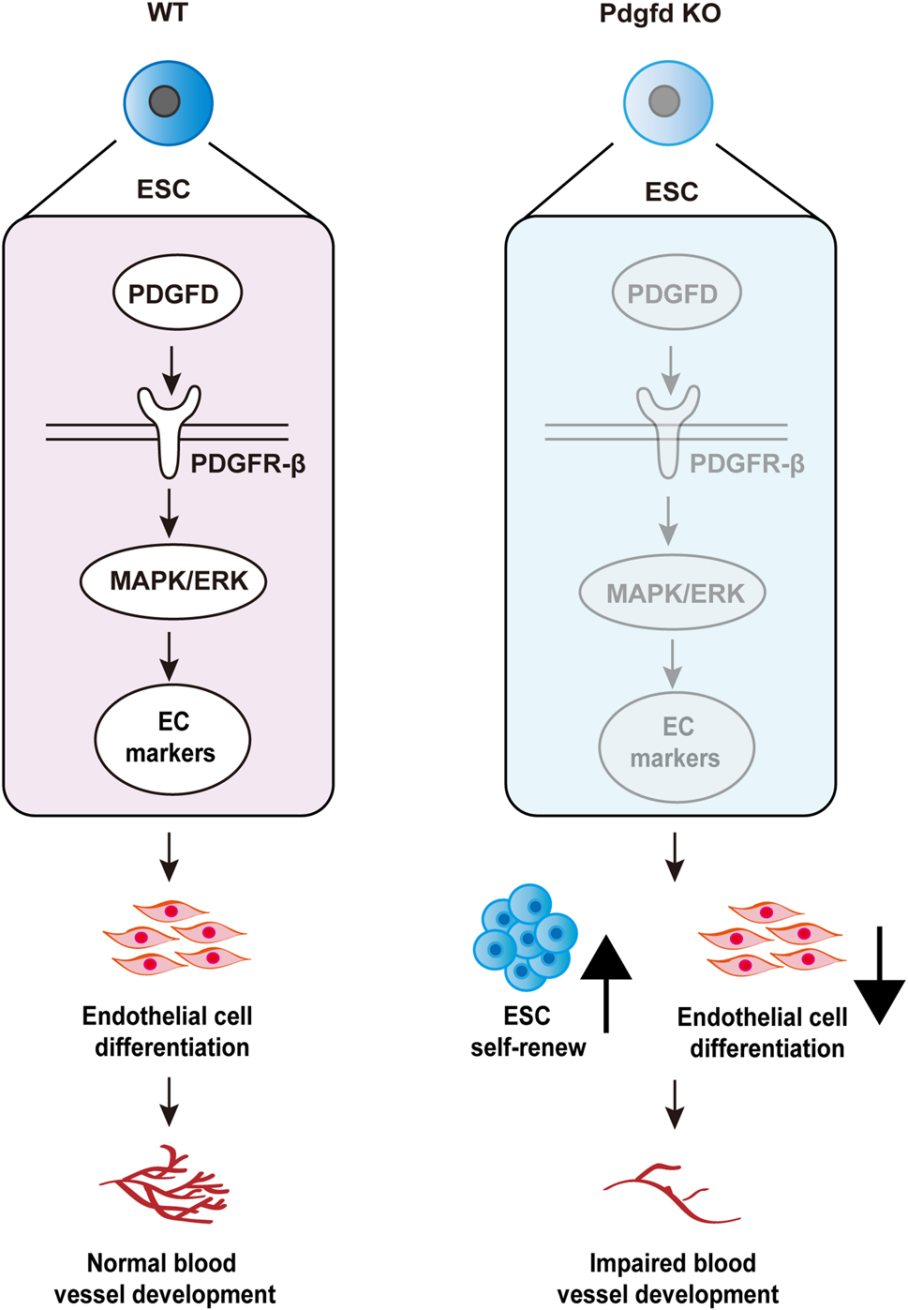
PDGFD promotes ESC vascular differentiation and represses ESC self-renewal by activating ERK signaling via PDGFR-β. Higher levels of PDGFD expression in ESCs activates PDGFR-β and ERK, resulting in increased expression of EC markers and decreased SOX2 expression, thus increased ESCs differentiation into endothelial cells and decreased ESCs self-renewal

In this study, we uncover an important role of PDGFD in promoting ESC endothelial differentiation and suppressing ESC self-renewal (Fig. 8). Genetic deletion of *Pdgfd* in mice impaired blood vessel formation in both embryonic development and teratoma growth, and overexpression of PDGFD-induced ESC differentiation towards EC lineage. Our findings suggest potential therapeutic implications of modulating PDGFD activity for stem cell therapy, particularly, for vascular diseases with EC defects.

Traditionally, the PDGF family is known for their effects on mesenchymal cells, mural cells and tumor growth [1, 4]. Studies on PDGFs and their receptors in relationship to stem cells are limited with diverse observations. PDGFA has been shown to induce astrocyte differentiation in human ESCs [21], while another study showed that low concentrations of PDGFA maintain the undifferentiated state of ESCs [22]. PDGFB has been reported to promote ESC differentiation into smooth muscle cells [23] or cardiomyocytes [24], while it has also been shown that PDGF-BB, together with S1P, prevents ESC differentiation [25]. Observations on the effects of the two PDGF receptors on stem cells also differ. It was reported that PDGFR-α induces ESC differentiation into mesendoderm cells [26] and PDGFR-β promotes ESC differentiation into smooth muscle cells [27] or myeloid lineage [28], while it has also been shown that the PDGF receptors prevent ESC differentiation [22]. These findings suggest that the effects of the PDGFs and their receptors on ESCs might be context- or cell type-specific. Thus far, the potential effect of PDGFD on ESCs remains unclear.

Here, we found that during ESC differentiation, PDGFD expression increased by almost 10-folds, indicating an important role of PDGFD in this process. Indeed, loss of PDGFD by shRNA knockdown inhibited ESC differentiation and increased ESC self-renewal in multiple assays, such as colony formation, EB formation, and teratoma formation assays, demonstrating a key role of PDGFD in ESC differentiation. Moreover, *Pdgfd* knockdown in ESCs markedly decreased the protein level of BRACHYURY, a mesoderm marker, suggesting that PDGFD mainly affects ESC mesoderm commitment. In human mesenchymal stem cells (MSCs), PDGFD was shown to promote cell proliferation and sustain MSC multipotency [29]. The reasons for the different effects of PDGFD on ESCs and MSCs remain unclear thus far and future studies are required to address this.

Our RNA sequencing analysis showed that PDGFD-induced ESCs to differentiate towards vascular EC lineage. Indeed, *Pdgfd* knockdown decreased the expression of genes related to endothelium development, EC differentiation, and angiogenesis, and PDGFD treatment increased the expression of endothelial precursor cell (EPC) markers in ESCs. Consistently, and importantly, in vivo, decreased blood vessel densities were found in *Pdgfd*^*−/−*^ (exon two of *Pdgfd* gene deleted) mouse embryos, neonatal mice and teratomas derived from sh*Pdgfd* ESCs. In a previously published work [6], the exon one of *Pdgfd* gene was replaced by a LacZ reporter gene and the *Pdgfd*^−/−^ mice displayed a slightly higher blood pressure and disorganized NG2^+^ pericytes in the cardiac vasculature [6]. In addition, PDGFD was also found to be mainly expressed by vascular ECs [6], which is consistent with our results. However, this study did not investigate embryonic or neonatal *Pdgfd*^−/−^ mice. Other studies have shown potent angiogenic effects of PDGFD [30, 31]. It would be important to know whether PDGFD-induced ECs could contribute to various physiological or pathological angiogenesis, and more studies are warranted to investigate into this.

The signaling pathway induced by PDGFD are not well understood thus far. In this work, RNA sequencing revealed that ERK signaling is a major pathway activated by PDGFD. PDGFD knockdown decreased ERK activation, and PDGFD protein treatment activated both ERK and PDGFR-β in ESCs. Furthermore, blocking the ERK pathway in ESCs completely abolished PDGFD-induced upregulation of EC markers, and increased SOX2 expression. ERK activation can promote ESC exit from pluripotency by regulating the phosphorylation of multiple transcription factors (TFs). For example, ERK activation phosphorylates NANOG and inhibits its transactivation by decreasing NANOG stability through ubiquitination [32]. Moreover, ERK activation initiates KLF4 nuclear export to the cytoplasm and leads to rapid ESC differentiation [33]. In ESCs, SOX2 harbors several putative ERK phosphorylation sites [34]. Yet, it remains unclear whether SOX2 activity or stability is regulated by ERK. In this study, we unveal that the nuclear fraction of SOX2 in ESCs decreased after ERK activation, thus raising the possibility that ERK might modulate SOX2 translocation in ESCs.

The observed effect of PDGFD on ESCs appeared to be specific, since PDGFB, the other PDGFRβ ligand, did not show the same effects in terms of ESC proliferation, inhibition of pluripotency genes, and EC marker induction. Indeed, these findings are consistent with previous reports showing different effects of PDGFD and PDGFB on adipose-derived stem cells [13]. We do not know the underlying mechanisms for the functional differences between PDGFD and PDGFB at this stage. Yet, multiple potential possibilities may exist. For example, the *PDGFD* and *PDGFB* genes use different enhancers. First, enhancer GH11J104162 regulates *PDGFD* but not *PDGFB* in ESCs and ECs (GeneCards). It is known that enhancer–promoter interactions can be stabilized and regulated by a group of proteins with similar functions [35]. The different protein-enhancer–promoter interactions might be involved in the functional aspects of PDGFD and PDGFB. Moreover, the *PDGFD* and *PDGFB* promoters have different TF binding sites (Table S1). Indeed, in mouse ESCs, ChIP-seq analysis (https://www.signalingpathways.org) revealed different TFs for *Pdgfd* and *Pdgfb* promoters (Table S2). It is known that different TFs may lead to distinct TF-dependent chromatin remodeling and gene expression [36, 37], and therefore different functional readouts. Further studies are needed to verify these.

In summary, our findings provide new insights into how EC commitment and renewal of ESCs are regulated by revealing PDGFD’s critical role in switching on ESC differentiation towards EC lineage while suppressing ESC renewal via the ERK pathway. Modulating PDGFD activity may have therapeutic potential in stem cell therapy for the treatment of various vascular diseases with EC defects.

## Materials and methods

### Mice

All mice were housed and maintained on a 12/12 h light/dark cycle at the Animal Facility of Zhongshan Ophthalmic Center, Sun Yat-Sen University. Six-week old nude mice were purchased from the GemPharmatech Company (Nanjing, China). *Pdgfd* deficient mice were generated by the Cyagen Company (Nanjing, China), and bred on a C57BL/6J background for more than six generations. Briefly, two gRNAs targeting *Pdgfd* exon 2 together with Cas9 mRNA were injected into mouse zygotes, which were subsequently transplanted into pseudopregnant mice. The neonatal mutant mice were genotyped by PCR and sequencing. The positive founder (F0) was bred to establish *Pdgfd*^+*/−*^ mouse line. The primers used for genotyping the *Pdgfd* deficient mice by PCR are 5′-ATAACATAGTAAAGCGAAAACATGAACTG-3′ and 5′-GGCCACTCTTGTGGAAGATAATCTC-3′. A 928 bp PCR product represents the wild-type mice, and a 457 bp band represents the deletion of the *Pdgfd* gene. The absence of *Pdgfd* exon 2 mRNA in *Pdgfd*^*−/−*^ mouse hearts (Fig. S5c) was confirmed by qRT-PCR. The primers are 5′-TTCCCGAACAGCTACCCAAG-3′ and 5′-TCCTTGGAGGGATCTCCTTGT-3′.

### Cell culture and treatment

Mouse ESCs E14 (Darwin Core Facility, Baylor College of Medicine) were maintained in culture dish coated with 0.1% gelatin (Sigma-Aldrich, St. Louis, USA) in ESC medium containing KO DMEM medium (Thermo Fisher Scientific, Gibco, Waltham, MA, USA) and supplemented with 15% (v/v) fetal bovine serum (FBS; Gibco, Waltham, MA, USA), β-mercaptoethanol (0.1 µM), GlutaMax-I supplement (2 mM), MEM non-essential amino acids (0.1 mM), 1% (v/v) Penicillin–Streptomycin, LIF (1000 U/mL, Millipore, Billerica, MA, USA), and 2 inhibitors (CHIR99021, PD325901 at 3 µM and 1 µM, respectively) (Hejian Technology Co., Suzhou, China). HEK293T cells (COBIOER, Nanjing, China) were maintained in DMEM medium (Sigma-Aldrich, St. Louis, USA) with 10% (v/v) FBS, 1% Penicillin–Streptomycin. Mouse embryonic fibroblasts (MEFs, Cyagen, Santa Clara, CA, USA) serves as feeder cells and were maintained in DMEM medium with 10% FBS and 1% Penicillin–Streptomycin. The MEFs were treated with Mytomycin C (10 µg/mL, Sigma-Aldrich, St. Louis, USA) for 3 h before culturing in ESC medium. All cells were cultured at 37 °C in humidified air with 5% CO_2_.

For PDGFD or PDGFB protein stimulation, ESCs were starved in serum-free medium overnight and then stimulated with 50 ng/mL PDGFD protein (R&D, Minneapolis, MN, USA) or PDGFB protein (PeproTech, Cranbury, NJ, USA) for indicated timepoints. For ERK inhibition, the MEK inhibitor PD0325901 (1 μM) was applied for 2 days in ESC medium without 2i. For TPA treatment, cells were starved in serum-free medium overnight followed by treatment with 200 nM TPA (New England Biolabs, Ipswich, MA, USA) for 15 or 30 min. For Crenolanib treatment, cells were starved in serum-free medium overnight, followed by treatment with 50 ng/mL PDGFD protein together with 100 nM Crenolanib (Cayman Chemical, Ann Arbor, Michigan, USA) or control goat IgG (R&D, Minneapolis, MN, USA) for 30 min. The cells were then collected for Western blot.

### ESC differentiation

For LIF withdrawal-induced ESC differentiation, ESCs were cultured in ESC medium in the absence of LIF for 3 days [38]. For RA-induced ESC differentiation, ESCs were cultured in ESCs medium containing 5 µM RA (Sigma-Aldrich, St. Louis, USA) for three days [39]. For embryoid body (EB)-induced ESC differentiation, EB formation was performed for 6 days (see detailed description below). For endothelium differentiation, 5 × 10^5^ ESCs were cultured in a 100 mm petri dish in the absence of LIF for 2 days and cultured further with 30 ng/mL recombinant human VEGFA protein (PeproTech, Cranbury, NJ, USA) for 7 days [40].

### Embryoid body (EB) formation

ESCs were seeded at a density of 4 × 10^4^ cells/mL using a hanging drop (25 µL) method and cultured without LIF (−LIF) in ESC medium for six days in 150 mm dishes. At day 6, all the EBs were harvested, photographed and analyzed [41].

### Detection of secreted PDGFD

Mouse ESCs were plated in two 10 cm dishes (1 × 10^6^ each) and cultured in ESC medium. The next day, one dish was refreshed with normal ESC medium while the other switched to ESC medium without LIF (−LIF). After 48 h, ESCs were rinsed with PBS and maintained in 5 mL serum-free ESC medium with or without LIF for 2 days. The medium were then concentrated using Amicon Ultra-4 centrifugal filter units (UFC801024) and analyzed by Western blot. Ponceau S staining was used as a loading control.

### PDGFB knockdown by siRNA

ESCs were transfected with siRNA oligos (GenePharma) targeting mouse PDGFB or scrambled siRNA as a control using Lipofectamine 2000 (Invitrogen). ESCs were trypsinized and 1 × 10^6^ cells were seeded in a 60 mm culture dish with 4 mL medium/well. For each well, 200 pmol of siRNA was mixed with 500 µL of Opti-MEM (Thermo), and 10 µL Lipofectamine 2000 (Invitrogen) was mixed with another 500 µL of Opti-MEM, and incubated for 5 min. The two different mixtures were subsequently combined and incubated for 15 min, and added into each well. After 6 h, the culture medium was changed with new medium. Forty-eight hours after transfection, the ESCs were harvested for analysis. The sequences of the PDGFB siRNA oligos are: sense, 5′-GCCUGCAAGUGUGAGACAGUA-3′, and antisense, 5′-UACUGUCUCACACUUGCAGGC-3′.

### PDGFR-β neutralization

ESCs were starved in serum-free medium overnight. The next day, ESCs were refreshed with serum-free medium and supplemented with 50 ng/mL PDGFD protein together with 0.6 μg/mL PDGFR-β receptor neutralizing antibody (Thermo Fisher Scientific, Invitrogen, Waltham, MA, USA) or control goat IgG (R&D, Minneapolis, MN, USA) for 30 min. The cells were then collected for Western blot analysis.

### Generation of stable *Pdgfd* knockdown or overexpression ESCs

To generate stable *Pdgfd* knockdown ESCs, 1 μg of pLent-*Pdgfd*-4in1-shRNA plasmids (Vigene Biosciences, Rockville, MD, USA) expressing shRNAs targeting four different mouse *Pdgfd* sequences were transfected into HEK293T cells together with 2 μg of lentivirus packing plasmids pSPAX2 and pMD2G (Addgene, Watertown, MA, USA) using Lipofectamine 2000 (Life Technologies, Waltham, MA, USA). The supernatants containing the viral particles was collected after 48 h and added to the ESCs in the presence of 5 μg/mL of polybrene (Santa Cruz, Dallas, TX, USA). After 6 h, new ESC medium was added. The ESCs were selected using puromycin (2 μg/mL). The pLent-4in1-shRNA-GFP-Puro plasmid (Vigene Biosciences, Rockville, MD, USA) was used as a negative control. The stable *pdgfd*-overexpressing ESCs were constructed following the same procedure. Mouse *pdgfd* gene sequence was inserted into the plent-EF1a-FH-CMV-GFP-P2A-puro plasmid (Vigene Biosciences, Rockville, MD, USA). PCR primers used are 5′-CG GGATCCCGATGCAACGGCTCGTTTTAGT-3′ and 5′-ATAGCGGCCGCTCGAGGTGGTCTTGAGCTG CAGAT-3′. The shRNA sequences used are listed in Table S3.

### Generation of ***Pdgfd***^−/−^ and ***Pdgfd***^+***/***+^ ESCs

*Pdgfd*^−/−^ and *Pdgfd*^+/+^ ESCs were derived from 3.5 day-old blastocyst-stage embryos obtained from *Pdgfd*^+/−^ heterozygous breeding. Blastocysts were collected by flushing of oviducts and uteri using M2 media (Sigma-Aldrich, St. Louis, USA). Embryos were collected to four-well plates (Thermo Fisher Scientific, Waltham, MA, USA) with feeder cells in ESC medium. After 6–12 days, the ICM outgrowth was re-plated after trypsinization using 0.25% trypsin (Invitrogen Corp., Grand Island, NY, USA) on four-well plates with feeder cells. When the ESCs were sub-confluent, they were plated into larger gelatin-coated culture dishes. The ESCs were passaged every 2–4 days and genotyped by PCR.

### Analysis of *Pdgfd* deficient embryos and neonatal mice

*Pdgfd* mutant embryos and neonatal mice were obtained from *Pdgfd*^+*/−*^  × *Pdgfd*^+*/−*^ breeding. Briefly, 6–8 weeks old female mice were superovulated by intraperitoneal injection of 5 IU pregnant mare’s serum gonadotropin (PMSG; Solarbio, Beijing, China), and after 48 h, followed by the injection of 5 IU human chorionic gonadotrophin (hCG; Millipore, Billerica, MA, USA). The female mice were then caged with male mice at a one-to-one ratio. The presence of a vaginal plug in a female mouse about 12–20 h after hCG injection marks 0.5 days post copulation.

For mouse blastocyst collection, embryos were flushed from oviducts and uteri using M2 media (Sigma-Aldrich, St. Louis, USA) at E3.5. The blastocysts were subjected to whole transcriptome amplification using the PEPLI-g WTA single cell kit (Qiagen, Hilden, Germany) following the manufacturer’s instructions. The product was 10-fold diluted, and 1 µL of the diluted product was used for qRT-PCR.

For E10.5 or E12.5 embryo collection, the embryos were dissected from uterus, washed with phosphate buffered saline (PBS) and then grinded in RIPA (Solarbio, Beijing, China) or Trizol Reagent (Invitrogen, Waltham, MA, USA) and subjected to Western blot or qRT-PCR.

### Colony formation assay and alkaline phosphatase (AP) staining

ESC colony formation assay was performed by plating the ESCs onto 0.1% gelatin-coated 6-well plates at different densities (200, 400, 800 cells per well) and culturing for seven days. Colony formation was analyzed by AP staining prior to imaging. AP staining was performed using the AP detection kit (Vector Laboratories, Burlingame, CA, USA) according to the manufacturer’s instructions.

### Cell proliferation assay

To analyze ESC proliferation, 1 × 10^4^ ESCs per well were plated in 12-well plates and cultured for 4 days. Cell numbers were quantified at 72 and 96 h using an automated cell counter (Inno-Alliance Biotech, Wilmington, DE, USA). Triplicate samples were used for each group at each time point.

### RNA isolation, cDNA synthesis and quantitative real-time PCR (qRT-PCR)

Total RNA was isolated using the Trizol reagent (Invitrogen, Waltham, MA, USA) following the manufacturer’s instructions. A total of 2 µg RNA was reverse transcribed to cDNA using the FastQuant RT Kit (Tiangen Biotech, Beijing, China), and then amplified with qRT-PCR via SYBR Green PCR Master Mix (Vazyme Biotech, Nanjing, China) using an ABI QuantStudio 6 Flex device (Life Technologies, Waltham, MA, USA). When the expressions of multiple genes were investigated, to show the overall expression levels, the expression of each gene was normalized against GAPDH using a ∆*C*_t_ method [∆*C*_t_ = *C*_t_ of the gene − *C*_t_ of GAPDH. Relative gene expression = $2^{{ - (\Delta C_{{\text{t}}} )}}$]. When the expression of only one gene was investigated, a ∆∆*C*_t_ method was used [∆∆*C*_t_ = ∆*C*_t_ of the gene − ∆*C*_t_ of control. Relative gene expression = $2^{{ - (\Delta \Delta C_{{\text{t}}} )}}$]. The sequences of the primers used are listed in Table S4.

### Cellular fractionation

To obtain different cellular fractionations, ESCs were lysed using cytoskeletal buffer (50 mM NaCl, 300 mM sucrose, 10 mM Pipes, pH 6.8, 3 mM MgCl_2_, 0.5% Triton X-100, protease inhibitor (Beyotime Biotechnology, Shanghai, China), 1 mM DTT, 1 mM PMSF) for 30 min on ice. After centrifugation for 10 min at 4 °C, the cytoplasmic fraction in the supernatant was collected. The nuclear fraction in the pellet was washed, lysed in RIPA buffer (Solarbio, Beijing, China) and collected.

### Western blot

Western blots were performed by separating proteins on SDS-PAGE gels followed by protein transfer to PVDF membranes (Bio-Rad). The antibodies used were: anti-PDGFD (sc-137030, Santa Cruz), anti-SOX2 (AF-2018, R&D), anti-BRACHYURY (sc-166962, Santa Cruz), anti-ERKs (4696, Cell Signaling Technology), anti-Phospho ERKs (4370, Cell Signaling Technology), anti-PDGFR-β (3169, Cell Signaling Technology), anti-Phospho PDGFR-β (Tyr1021) (2227, Cell Signaling Technology), anti-PECAM1 (222783, Abcom), anti-Phospho STAT3 (9145, Cell Signaling Technology), anti-STAT3 (9139, Cell Signaling Technology), anti-KDR (9698, Cell Signaling Technology), anti-HSP90 (7411, Cell Signaling Technology), anti-TUBULIN (RM2007, Ray Antibody), anti-β-ACTIN (RM2001, Ray Antibody), anti-GAPDH (70-Mab5465-040, MultiSciences), anti-HISTONE 3 (GB13102-1, Servicebio), Goat anti-mouse IgG (GAM0072, MultiSciences), Goat anti-rabbit IgG (GAR0072, MultiSciences), Rabbit anti-goat IgG (RAG0072, MultiSciences), anti-PDGFB (PA1-27394, Invitrogen), anti-uPA (17968-1-AP, Proteintech) and anti-PLASMIN (66399-1-Ig, Proteintech). The bands were visualized using a GBOX-CHEMI-XX8 device (SYNGENE).

### Teratoma formation and immunohistochemistry analysis

The sh*Control* or sh*Pdgfd* ESCs (1 × 10^6^ cells/100 µL PBS) were injected subcutaneously into the dorsal flank regions of nude mice. Four weeks after injection, the teratomas were surgically dissected and weighed. Parts of the tumors were used for RNA extraction, and the rest were fixed in 4% paraformaldehyde, embedded in paraffin and sectioned (5 µm) for immunohistochemistry (IHC) staining.

For IHC staining, sections were placed at 60 °C for 1 h followed by deparaffinized with xylene and ethanol. Subsequently, the sections were processed in 10 mM citrate buffer (pH 6.0) and boiled for 10 min for antigen retrieval. After cooling in room temperature, the sections were incubated in 3% hydrogen peroxide for 10 min to block the endogenous peroxidase activity. The sections were stained with anti-Collagen IV (BioRad, Hercules, California, USA) overnight at 4 °C and then incubated using the Elivision kit (Maxim Biotech, Fuzhou, China) for 30 min at room temperature. The DAB color reagent (Maxim Biotech, Fuzhou, China) was used. Finally, hematoxylin staining was performed for nuclear visualization. Sections were visualized using a fluorescence microscope-Axio Imager Z2 (ZEISS, Oberkochen, Germany). ImageJ software was used to analyze the sections. Collagen IV density was calculated as Collagen IV^+^ pixels/total field pixels.

### Immunofluorescence analysis

E12.5 mouse embryos or the hearts from P1 pups were fixed in 4% paraformaldehyde (Sigma-Aldrich, St. Louis, USA) overnight at 4 °C, and then transferred into 30% sucrose overnight at 4 °C. The tissues were then embedded in Tissue-Tek O.C.T. compound (Sakura Finetek, Tokyo, Japan) and sectioned. The frozen sections were placed in room temperature for 30 min, blocked with 5% donkey serum, 0.5% Triton X-100 in 1 × PBS for one hour at room temperature. The sections were stained with anti-PECAM1 (BD Biosciences, New Jersey, USA) overnight at 4 °C and then stained with fluorescein-conjugated secondary antibodies (Life Technologies, Waltham, MA, USA) for 1 h at room temperature. Sections were visualized using a fluorescence microscope-Axio Imager Z2 (ZEISS, Oberkochen, Germany). All sections were imaged using a × 20 objective. The ImageJ software was used for image analysis. The ventricle areas of the hearts were imaged and analyzed. Vascular density was calculated as PECAM1+ pixels/total field pixels.

### RNA sequencing (RNA-seq)

Total RNA was extracted from shControl or shPdgfd ESCs (triplicates for each group) using Trizol reagent (Invitrogen, Waltham, MA, USA). RNA-seq was performed using an Illumina Nova Seq 6000 sequenator (Illumina, San Diego, CA, USA) by Kangcheng Bio-tech, Inc. (Shanghai, China). The differentially expressed genes (DEGs) were defined at *p* < 0.05 and fold change > 1.5. The DEGs were subjected to GO analysis using the topGO package for enriched biological processes, and the KEGG pathway analysis using Fisher’s exact test for enriched pathways (*p* < 0.05). GSEA enrichment plots were generated using the GSEA software [42]. Default parameters were used for GSEA analysis. The RNA sequencing data were accessible in GEO database (GSE172117).

### Statistical analysis

GraphPad Prism (GraphPad Software, Inc.) was used for statistical analyses except for the RNA-seq data. All data are presented as mean ± SD. For comparisons between two groups, two-tailed Student’s *t* tests were used. For comparison among more than two groups, one-way ANOVA analysis was used. *N* numbers, *p* values and other detailed information are provided in the corresponding figure legends.

## Supplementary Information

Below is the link to the electronic supplementary material.

Supplementary file1 (DOCX 1854 kb)

## Data Availability

The data sets generated during the current study are available in the GEO database (GSE172117).
