# Supplementary material for: PDGFD switches on stem cell endothelial commitment
Source: Angiogenesis. 2022 Jul 20;25(4):517–33. doi: 10.1007/s10456-022-09847-4 (PMC9519648; doi:10.1007/s10456-022-09847-4)
Supplement: Supplementary file 1 — Supplementary file1 (DOCX 1854 kb) [file 10456_2022_9847_MOESM1_ESM.docx]

Supporting Information

**PDGFD switches on stem cell endothelial commitment**

Weisi Lu^1^*†, Peipei Xu^1^†, Boxiong Deng^1^†, Jianing Zhang^1^†, Ying Zhan^1^, Xianchai Lin^1^, Xiangzhong Xu^2^, Zhaoxia Xia^3^, Xiaoxi Yang^2^, Xiaoling Zeng^1^, Lijuan Huang^1^, Bingbing Xie^1^, Chenghu Wang^2^, Shasha Wang^1^, Haiqing Kuang^1^, Xianjing Han^1^, Antonio Mora^4^, Yihai Cao^5^, Qin Jiang^2^*, Xuri Li^1^*

^1^State Key Laboratory of Ophthalmology, Zhongshan Ophthalmic Center, Sun Yat-sen University, Guangdong Provincial Key Laboratory of Ophthalmology and Visual Science, Guangzhou 510060, China.

^2^Affiliated Eye Hospital of Nanjing Medical University, Nanjing 210000, China
^3^Department of Ophthalmology, The Sixth Affiliated Hospital of Sun Yat-sen University, Guangzhou, China.

^4^Joint School of Life Sciences, Guangzhou Medical University and Guangzhou Institutes of Biomedicine and Health (Chinese Academy of Sciences), Guangzhou, China

^5^Department of Microbiology, Tumor and Cell Biology, Karolinska Institute, 17177 Stockholm, Sweden

*Corresponding authors:

Xuri Li, [lixr6@mail.sysu.edu.cn](mailto:lixr6@mail.sysu.edu.cn)

Weisi Lu, [luweisi3@mail.sysu.edu.cn](mailto:luweisi3@mail.sysu.edu.cn)

Qin Jiang, [jqin710@vip.sina.com](mailto:jqin710@vip.sina.com)

†These authors contributed equally to this work.

**
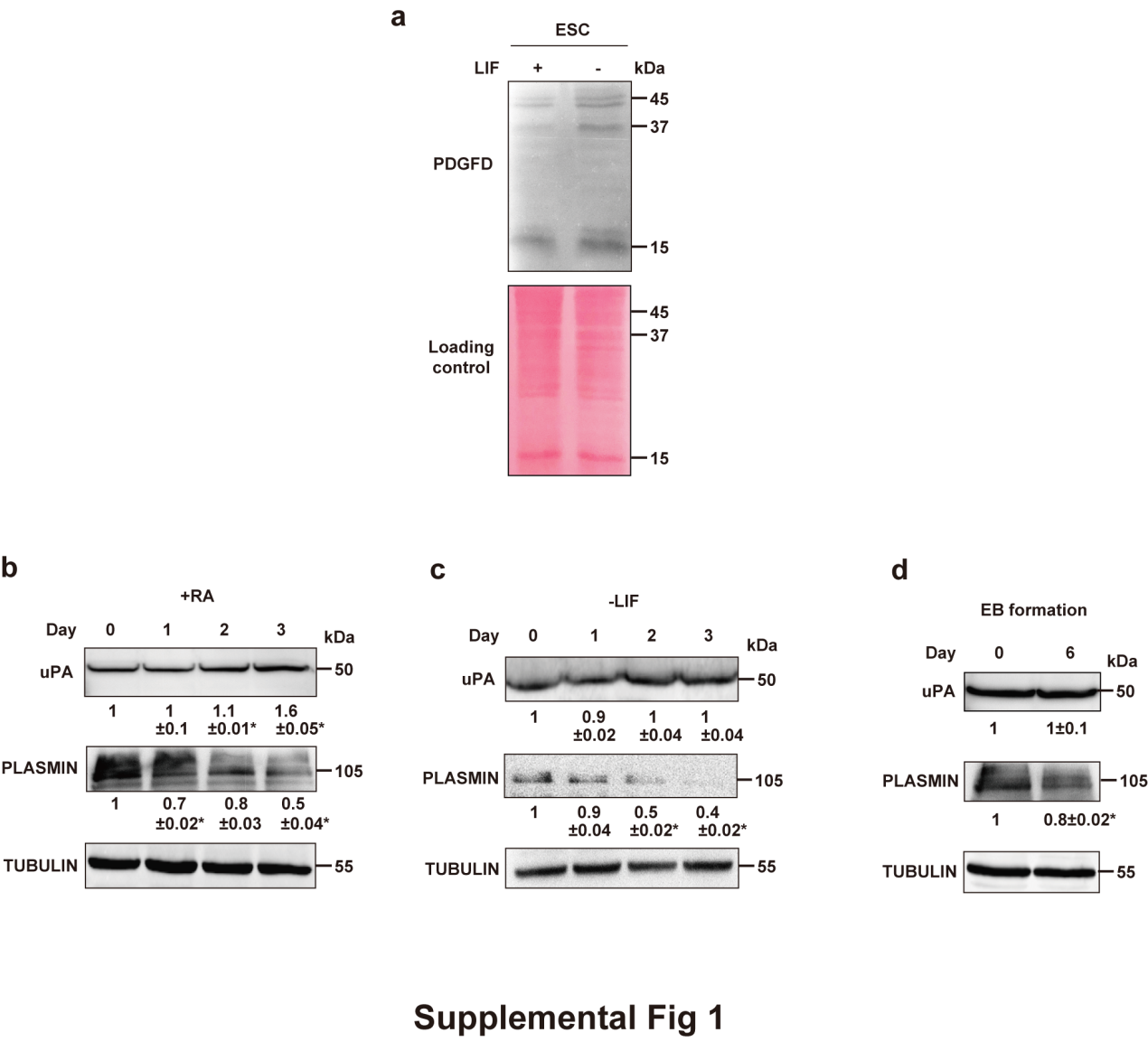
**

**Supplemental Fig. 1. PDGFD is proteolytically cleaved and expression of PLASMIN or uPA in ESCs during differentiation**

(**a**) Cleaved forms of PDGFD were detected in ESC-conditioned serum-free medium with or without LIF by Western blot (upper panel). Ponceau S staining was used as a loading control (lower panel).

(**b-d**) Both PLASMIN and uPA were detected by Western blot during ESC differentiation under various conditions. Densitometric quantification normalized to Tubulin in and expressed relative to Day 0 is shown beneath the blots. Data are presented as mean ± SD, n = 3. Statistical significance was determined using one-sample *t-test*. **p* < 0.05.

**
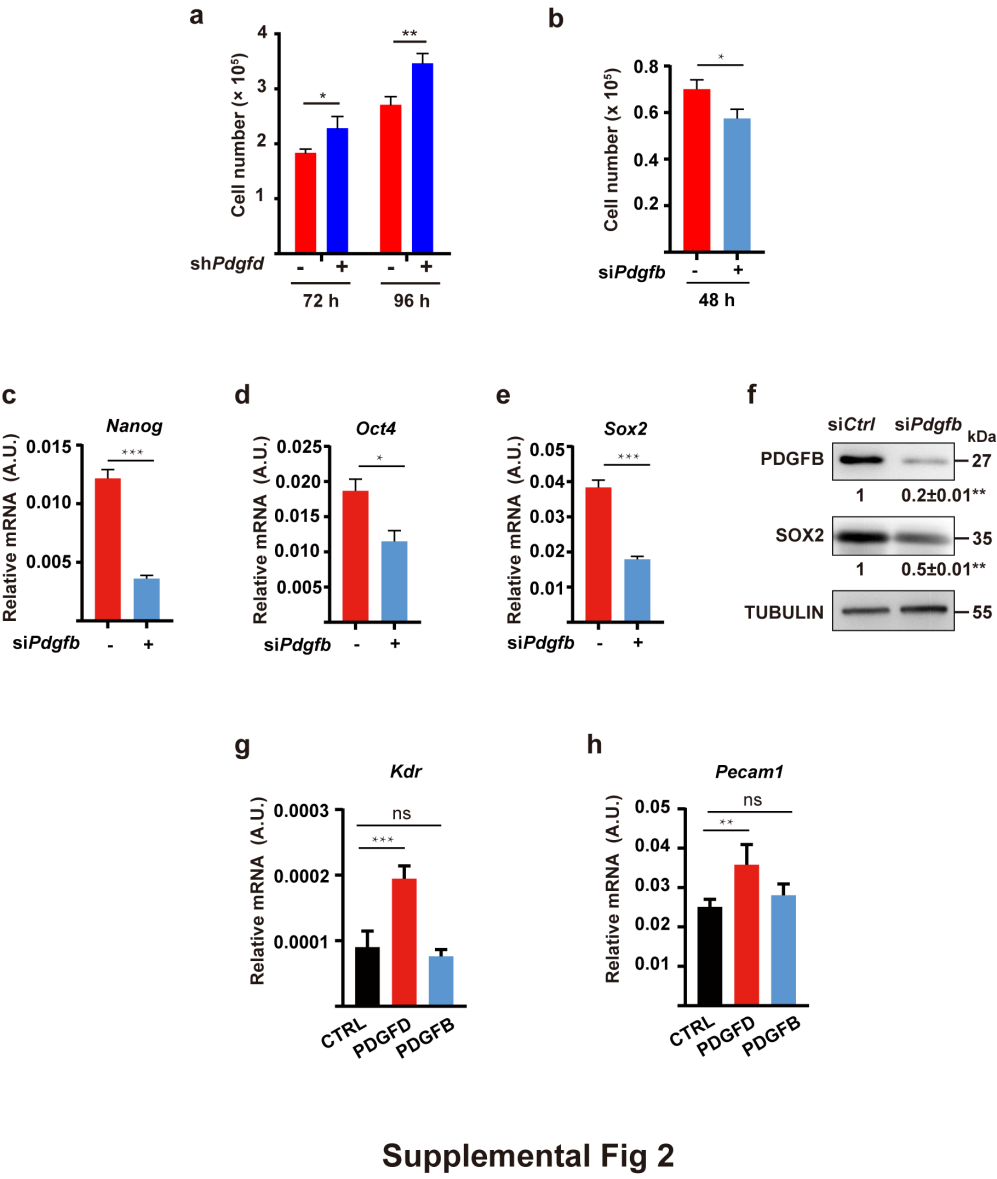
**

**Supplemental Fig. 2. Distinct effects of PDGFB on ESCs compared with PDGFD**

(**a**) Results showing that PDGFD knockdown increased ESC proliferation.

(**b**) Results showing that PDGFB knockdown decreased ESC proliferation.

(**c-f**) qRT-PCR (c-e) and Western blot (f) showing that PDGFB knockdown decreased the expression of the pluripotency markers Nanog, Oct4 and Sox2.

(**g,h**) qRT-PCR results showing that PDGFD treatment increased EC markers while PDGFB did not display such an effect. Densitometric quantification normalized to Tubulin in f and expressed relative to the control (siCtrl) is shown beneath the blots. Data are presented as mean ± SD, n = 3 each group. Statistical significance was determined using Student’s *t*-test in a-e, one-sample *t-test* in f, and one-way ANOVA in g-h. **p* < 0.05, ***p* < 0.01, ****p* < 0.001, ns: *p* > 0.05.

**
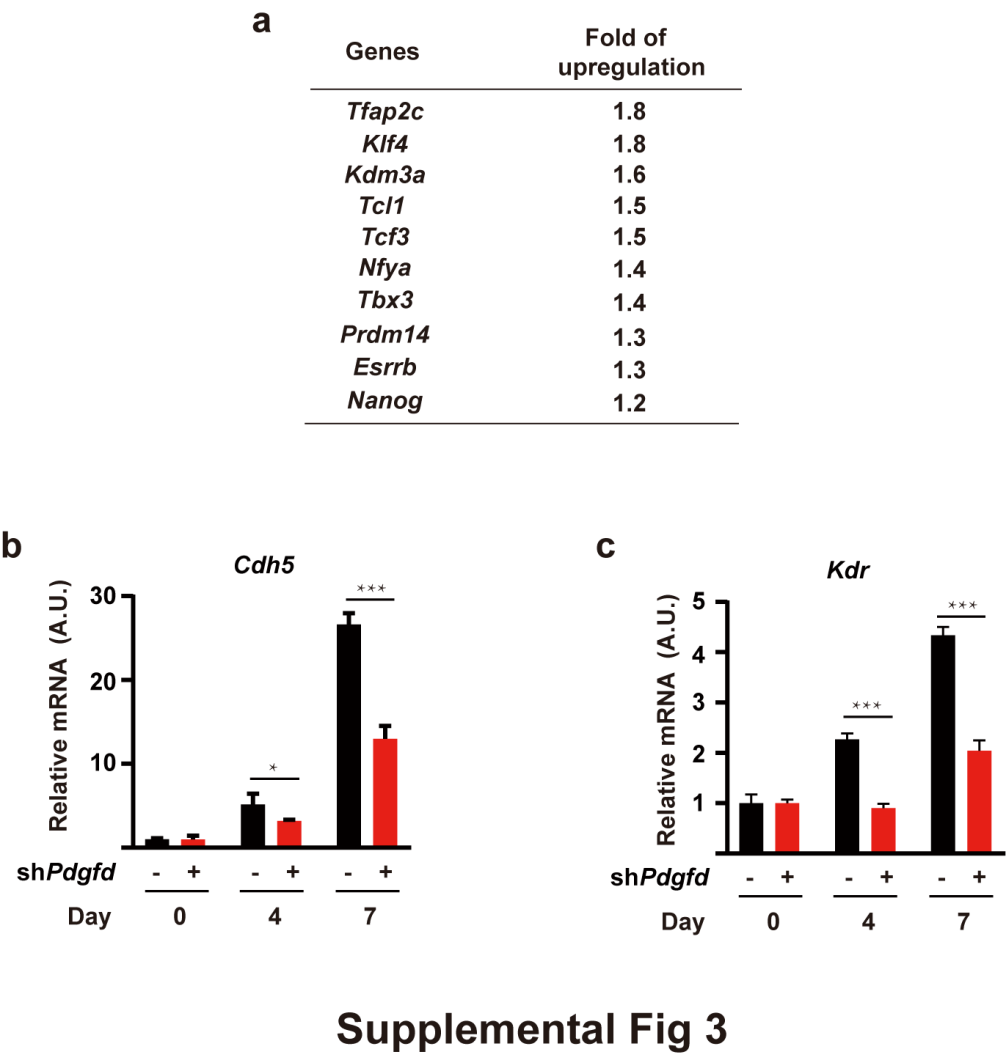
**

**Supplemental Fig. 3.** ***Pdgfd* knockdown up-regulated pluripotency genes and down-regulated EC markers.**

(a) RNA sequencing using sh*Pdgfd* ESCs showed that many of the up-regulated genes are related to ESC pluripotency.

(b,c) *Pdgfd* knockdown dampened the expression of *Cdh5* and *Kdr* in a VEGFA-induced EC differentiation assay. Data are presented as mean ± SD, n = 3 each group. Statistical significance was determined using Student’s *t*-test in b-c. **p* < 0.05, ****p* < 0.001.

**
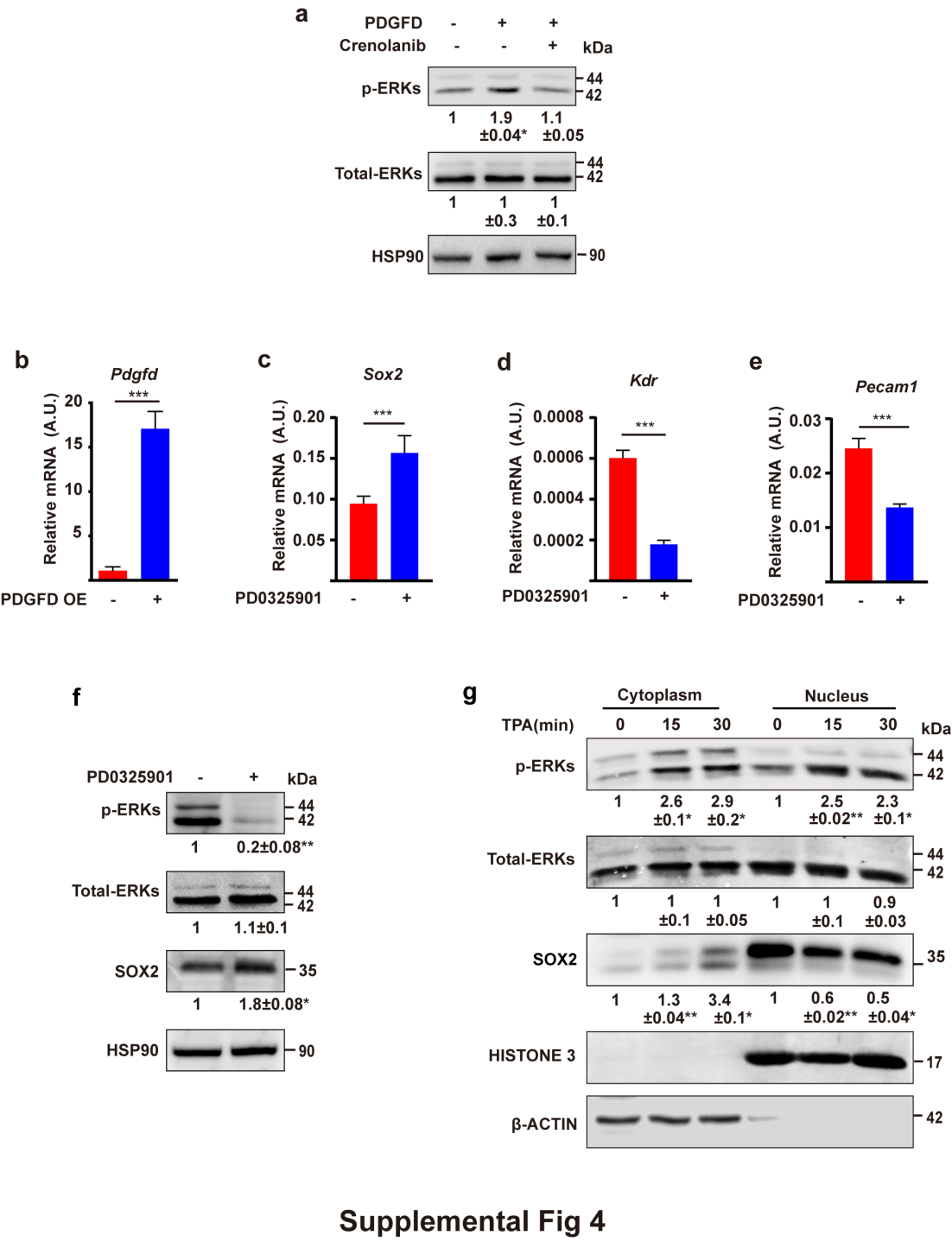
**

**Supplemental Fig. 4.** **PDGFD regulates ESC endothelial differentiation via ERK signaling.**

(**a**) Western blot showing that treatment of the ESCs with the PDGF receptor inhibitor Crenolanib abolished PDGFD-induced ERK phosphorylation.

(**b**) qRT-PCR results showing *Pdgfd* expression in PDGFD-overexpressing (PDGFD OE) ESCs.

(**c-e**) qRT-PCR results showing the expression of SOX2 and EC markers in ESCs treated with PD0325901.

(**f**) Western blot showing the expression of SOX2 and phosphorylated ERK in ESCs treated with PD0325901.

(**g**) Western blot results showing phosphorylated ERKs and SOX2 levels in ESCs treated with TPA. HISTONE 3 and β-ACTIN were used as nuclear or cytoplasmic controls respectively.

Densitometric quantification normalized to HSP90 (a, f) or HISTONE 3 and β-ACTIN (g) and expressed relative to Control group is shown beneath the blots. Data are presented as mean ± SD, n = 3 each group. Statistical significance was determined using Student’s *t*-test in b-e and one-sample *t-test* in a, f, g. **p* < 0.05, ***p* < 0.01, ****p* < 0.001.

**
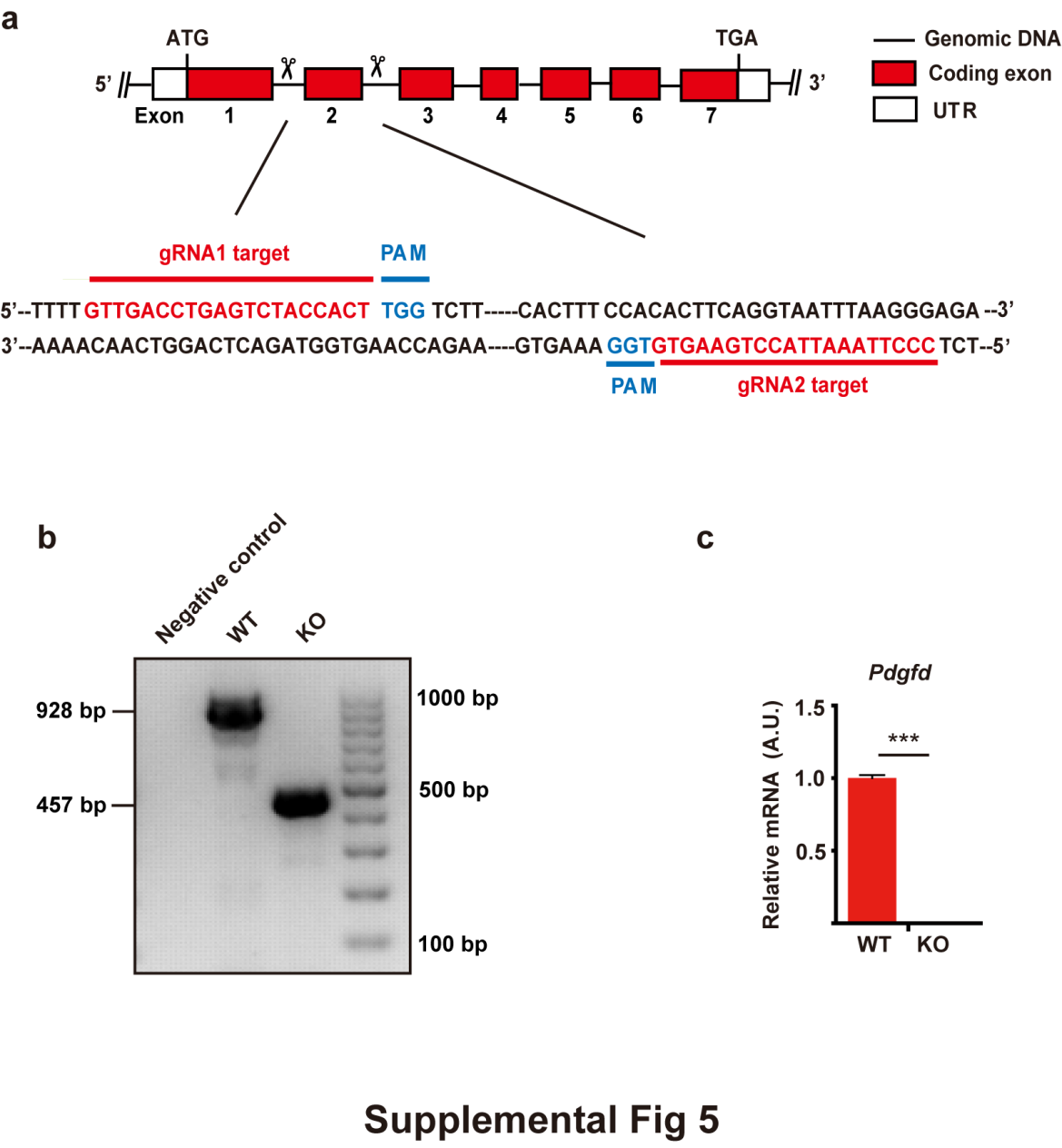
**

**Supplemetnal Fig. 5. Generation of *Pdgfd* deficient mice.**

(**a**) Diagram showing the strategy of generating *Pdgfd* knockout (KO) mice using CRISPR/Cas9 technology. Two gRNAs targeting the *Pdgfd* gene were used. ATG: start codon, TGA: stop codon, PAM: protospacer adjacent motif, UTR: untranslated region.

(**b**) PCR genotyping result of *Pdgfd* KO and WT mice.

(**c**) qRT-PCR analysis of *Pdgfd* expression in *Pdgfd*^+/+^ and *Pdgfd^-/-^* mouse hearts. Data are presented as mean ± SD, n = 3 each group. Statistical significance was determined using Student’s *t*-test in c. ****p* < 0.001.

**
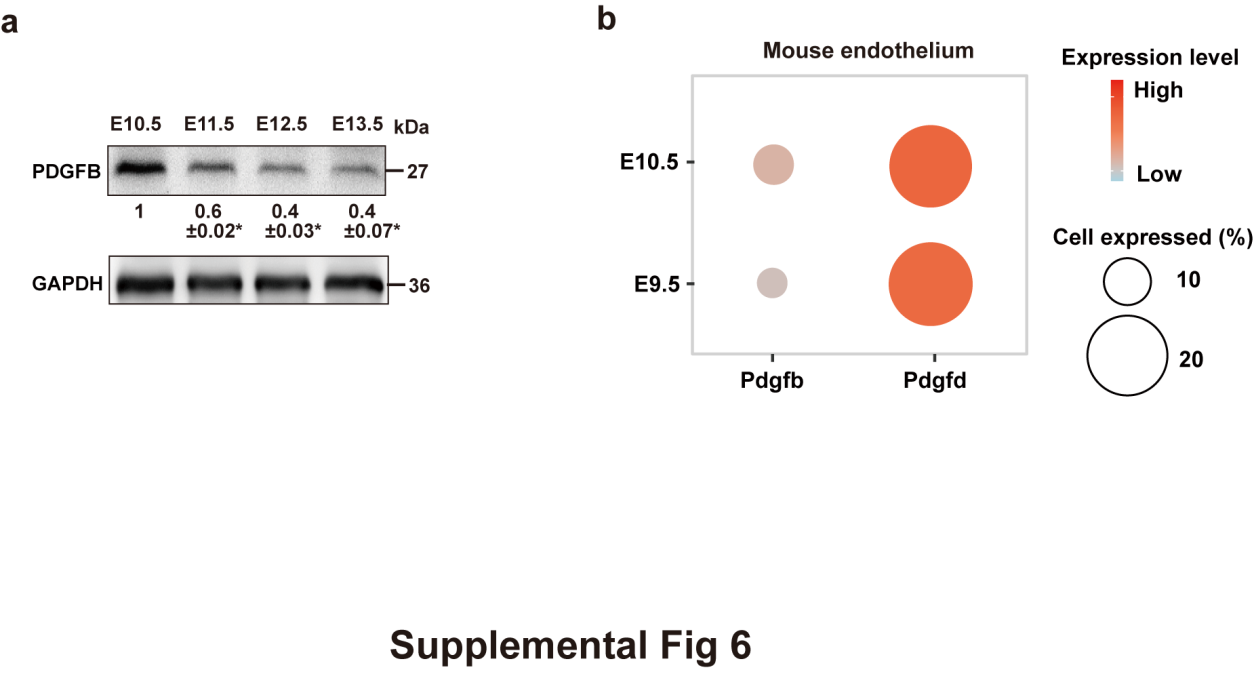
**

**Supplemetnal Fig. 6. PDGFB expression in mouse embryos**

(**a**) The protein levels of PDGFB decreased in E10.5-E13.5 mouse embryos. Densitometric quantification normalized to GAPDH and expressed relative to E10.5 is shown beneath the blots. Data are presented as mean ± SD, n = 3 each group. Statistical significance was determined using one-sample *t-test*. **p* < 0.05.

(**b**) scRNA-seq analysis of E9.5-10.5 mouse embryos revealed that PDGFD expression levels are markedly higher than those of PDGFB in endothelium.

**Table S1. Transcription factor binding sites in human *PDGFD* and *PDGFB* promoters**

| **Human *PDGFD* promoter** | **Human *PDGFB* promoter** |
| --- | --- |
| aMEF-2, AREB6, En-1, MEF-2A, MRF-2, Msx-1, NF-1, NF-1/L, Nkx2-5, TBP | NF-1, SRF, SRF (504 AA) |

**Table S2. Transcription factors binding to *Pdgfd* or *Pdgfb* promoters in mouse ESCs**

| **TFs binding to mouse *Pdgfd* promoter** | **TFs binding to mouse *Pdgfb* promoter** |
| --- | --- |
| Suz12, Rnf2, Zic2 | Suz12, Aff3, Ezh2, Pcgf2, Hcfc1, Kdm1a |

Table S3. shRNA sequences targeting *Pdgfd*

| **sh*Pdgfd*** | **Sequence** | |
| --- | --- | --- |
| shRNA-1 | | GCGCGAACTTTAGCTGCTATCTTCAAGAGAGATAGCAGCTAAAGTTCGCGCTTTTTT |
| shRNA-2 | | GCAATCACCTCACAGACTTGTTTCAAGAGAACAAGTCTGTGAGGTGATTGCTTTTTT |
| shRNA-3 | | GGATAACGTCAAGAACAAACCTTCAAGAGAGGTTTGTTCTTGACGTTATCCTTTTTT |
| shRNA-4 | | GGCAAGATGATCTGGAGAATTTTCAAGAGAAATTCTCCAGATCATCTTGCCTTTTTT |

**Table S4. qRT-PCR primers**

| **Gene** | **Forward primer** | **Reverse primer** |
| --- | --- | --- |
| *Pdgfd* | TACAGTTGCACTCCCAGGAAT | CTTCCAGTTGACAGTTCCGCA |
| *Gapdh* | TCCCACTCTTCCACCTTCGATGC | GGGTCTGGGATGGAAATTGTGAGG |
| *Nanog* | AGGCTTTGGAGACAGTGAGGTGC | TACCCTCAAACTCCTGGTCCTTC |
| *Nestin* | CTGCAGGCCACTGAAAAGTTC | TCTGACTCTGTAGACCCTGCTTCTC |
| *Gata6* | ACAGCCCACTTCTGTGTTCCC | GTGGGTTGGTCACGTGGTACAG |
| *Gata4* | CCCTACCCAGCCTACATGG | ACATATCGAGATTGGGGTGTCT |
| *Sox17* | AAGAAACCCTAAACACAAACAGCG | TTTGTGGGAAGTGGGATCAAGAC |
| *Sox1* | CCTCGGATCTCTGGTCAAGT | GCAGGTACATGCTGATCATCTC |
| *Brachyury* | CTCTAATGTCCTCCCTTGTTGCC | TGCAGATTGTCTTTGGCTACTTTG |
| *Oct4* | GCAGGAGCACGAGTGGAAAGCAAC | CAAGGCCTCGAAGCGACAGATG |
| *Sox2* | CGAGATAAACATGGCAATCAAATG | AACGTTTGCCTTAAACAAGACCAC |
| *Eomes* | GCGCATGTTTCCTTTCTTGAG | GGTCGGCCAGAACCACTTC |
| *Hand1* | GGCAGCTACGCACATCATCA | CCTGGCATCGGGACCATAG |
| *Gbx2* | AACTCAGCGAGGTGCAAGTAA | GGATGGGGACGACAATCTTGG |
| *Mia* | GTTCAGGGAGGTTACTATGGAGA | AGAAATCCCATTGATCGGTCTTC |
| *Nsd2* | TGCCAAAAAGGAGTACGTGTG | CTTCGGGAAAGTCCAAGGCAG |
| *Agpat1* | TAAGATGGCCTTCTACAACGGC | CCATACAGGTATTTGACGTGGAG |
| *Ncoa6* | GGAATCAACTTGGACACCACAGG | GACATGCTGCATGAGCTGAGGA |
| *Ang-1* | CACATAGGGTGCAGCAACCA | CGTCGTGTTCTGGAAGAATGA |
| *Pecam1* | GACGCCTTCATCCGAGTCG | CGGCCTCTTTGTCAGCTTTAGA |
| *Cdh5* | CACTGCTTTGGGAGCCTTC | GGGGCAGCGATTCATTTTTCT |
| *Kdr* | TTTGGCAAATACAACCCTTCAGA | GCAGAAGATACTGTCACCACC |
| *Vegfa* | GCACATAGAGAGAATGAGCTTCC | CTCCGCTCTGAACAAGGCT |
| *Tek* | CGGCCAGGTACATAGGAGGAA | TCACATCTCCGAACAATCAGC |
